# Impact of genetic risk and lifestyles on cardiovascular disease-free and total life expectancy: a cohort study

**DOI:** 10.1186/s13073-025-01487-9

**Published:** 2025-07-22

**Authors:** Dong Sun, Qiufen Sun, Yinqi Ding, Canqing Yu, Dianjianyi Sun, Yuanjie Pang, Pei Pei, Ling Yang, Iona Y. Millwood, Robin G. Walters, Huaidong Du, Jun Zhang, Dan Schmidt, Junshi Chen, Zhengming Chen, Liming Li, Jun Lv, the China Kadoorie Biobank Collaborative Group, Canqing Yu, Dianjianyi Sun, Pei Pei, Ling Yang, Iona Y. Millwood, Robin G. Walters, Huaidong Du, Jun Zhang, Dan Schmidt, Junshi Chen, Zhengming Chen, Liming Li, Jun Lv, Robert Clarke, Rory Collins, Richard Peto, Daniel Avery, Maxim Barnard, Derrick Bennett, Lazaros Belbasis, Ruth Boxall, Ka Hung Chan, Yiping Chen, Charlotte Clarke, Johnathan Clarke, Robert Clarke, Ahmed Edris Mohamed, Hannah Fry, Simon Gilbert, Pek Kei Im, Andri Iona, Maria Kakkoura, Christiana Kartsonaki, Hubert Lam, Kuang Lin, James Liu, Mohsen Mazidi, Sam Morris, Qunhua Nie, Alfred Pozarickij, Maryanm Rahmati, Paul Ryder, Saredo Said, Becky Stevens, Iain Turnbull, Baihan Wang, Lin Wang, Neil Wright, Xiaoming Yang, Pang Yao, Xiao Han, Can Hou, Qingmei Xia, Chao Liu, Lang Pan, Zengchang Pang, Ruqin Gao, Shanpeng Li, Haiping Duan, Shaojie Wang, Yongmei Liu, Ranran Du, Yajing Zang, Liang Cheng, Xiaocao Tian, Hua Zhang, Yaoming Zhai, Feng Ning, Xiaohui Sun, Feifei Li, Silu Lv, Junzheng Wang, Wei Hou, Wei Sun, Shichun Yan, Xiaoming Cui, Chi Wang, Zhenyuan Wu, Yanjie Li, Quan Kang, Huiming Luo, Tingting Ou, Xiangyang Zheng, Zhendong Guo, Shukuan Wu, Yilei Li, Huimei Li, Ming Wu, Yonglin Zhou, Jinyi Zhou, Ran Tao, Jie Yang, Jian Su, Fang Liu, Yihe Hu, Yan Lu, Liangcai Ma, Aiyu Tang, Shuo Zhang, Jianrong Jin, Jingchao Liu, Mei Lin, Zhenzhen Lu, Lifang Zhou, Changping Xie, Jian Lan, Tingping Zhu, Yun Liu, Liuping Wei, Liyuan Zhou, Ningyu Chen, Yulu Qin, Sisi Wang, Xianping Wu, Ningmei Zhang, Xiaofang Chen, Xiaoyu Chang, Mingqiang Yuan, Xia Wu, Xiaofang Chen, Wei Jiang, Jiaqiu Liu, Qiang Sun, Faqing Chen, Xiaolan Ren, Caixia Dong, Hui Zhang, Enke Mao, Xiaoping Wang, Tao Wang, Xi Zhang, Kai Kang, Shixian Feng, Huizi Tian, Lei Fan, XiaoLin Li, Huarong Sun, Pan He, Xukui Zhang, Min Yu, Ruying Hu, Hao Wang, Xiaoyi Zhang, Yuan Cao, Kaixu Xie, Lingli Chen, Dun Shen, Xiaojun Li, Donghui Jin, Li Yin, Huilin Liu, Zhongxi Fu, Xin Xu, Hao Zhang, Jianwei Chen, Yuan Peng, Libo Zhang, Chan Qu

**Affiliations:** 1 Department of Epidemiology & Biostatistics, School of Public Health, Peking University, Beijing, China; 2 Peking University Center for Public Health and Epidemic Preparedness & Response, Beijing, China; 3 Key Laboratory of Epidemiology of Major Diseases (Peking University), Ministry of Education, Beijing, China; 4 Clinical Trial Service Unit and Epidemiological Studies Unit (CTSU), Nuffield Department of Population Health, University of Oxford, Oxford, UK; 5 Suzhou Center for Disease Prevention and Control, Suzhou, China; 6 China National Center for Food Safety Risk Assessment, Beijing, China; 7 State Key Laboratory of Vascular Homeostasis and Remodeling, Peking University, Beijing, China

**Keywords:** Polygenic risk score, Lifestyles, Cardiovascular disease-free life expectancy, Cohort study

## Abstract

**Background:**

Understanding the role of genetic risk and lifestyles on life expectancy (LE) without cardiovascular disease (CVD) and total LE may help optimize healthy aging strategies after taking genetic background into account.

**Methods:**

The China Kadoorie Biobank recruited participants from five urban and five rural areas across China during 2004–2008 and followed them up till December 31, 2018. A polygenic risk score (PRS) comprising 3.5 million genetic variants for overall CVD was constructed by combining multiple PRSs for CVD and CVD-related risk factors in 96,400 participants. Genetic risk was categorized into low, intermediate, and high according to the PRS, and lifestyles were categorized as favorable, intermediate, and unfavorable according to the number of unfavorable lifestyles. Using multistate life tables, we estimated CVD-free and total LE at age 40 for different genetic and lifestyle risk groups.

**Results:**

Genetic risk was more strongly associated with CVD onset than post-CVD mortality. As a result, the increase in LE without CVD associated with low genetic risk (4.9 years (95% CI 4.3–5.5) for women and 4.4 years (3.6–5.1) for men) was greater than the increase in total LE (2.9 years (1.8–3.8) for women and 2.6 years (1.5–3.5) for men) when compared to high genetic risk. In contrast, the association strengths of lifestyles with CVD onset and mortality after CVD were similar. Correspondingly, compared to those with unfavorable lifestyles, participants with favorable lifestyles had longer total LE and LE without CVD of 3.0 (1.5–4.3) and 4.0 (3.0–4.9) years in women and 5.7 (4.1–7.1) and 5.8 (4.7–6.9) years in men, respectively. Participants with high genetic risk benefited more from favorable lifestyles than those with low and intermediate genetic risk, gaining 5.9 (2.3–9.3) and 5.3 (3.0–7.6) years in women and 6.1 (0.8–10.6) and 6.2 (2.3–9.8) years in men for total and CVD-free LE, respectively.

**Conclusions:**

Improving lifestyles is critical for reducing CVD-related healthcare burden and promoting healthy aging, especially for individuals with high genetic risk.

**Supplementary Information:**

The online version contains supplementary material available at 10.1186/s13073-025-01487-9.

## Background

With the irreversible aging trend, achieving healthy aging has become a global goal [1]. Healthy longevity entails not only living longer but also aging without major chronic disease and/or disability, resulting in lower healthcare costs and a reduced need for long-term care. Cardiovascular disease (CVD) is one of the major contributors to mortality and reduced quality of life globally, and its burden continues to increase [2, 3]. Although genetics may predispose certain adults to CVD and its risk factors, they do not act alone [4]. Adherence to healthy lifestyles has been associated with a significantly lower risk of coronary artery disease (CAD), stroke, and atrial fibrillation, regardless of the genetic risk category [5–8]. In comparison to both relative and absolute risk measures of CVD risk, life expectancy (LE) free of CVD and total LE are easier to understand for the lay public and policymakers, and can better demonstrate the impacts of genetic risk and lifestyles on public health burden caused by CVD and healthy aging [9, 10]. One study from the Atherosclerosis Risk in Communities (ARIC) study demonstrated an ideal Life’s Simple 7 (LS7) score was associated with longer coronary heart disease (CHD)-free and overall survival years across genetic risk groups [6].

A recent study revealed that the population attributable fractions of aggregate modifiable metabolic and behavioral risk factors for CVD and mortality risk varied by geographic region [11]. As a result, it is unclear whether the observed interplay between genetic risk and lifestyles for CHD-free and overall survival years in ARIC applies to the Chinese population. More importantly, a high CHD genetic risk has been linked to increased risks of the other CVD subtypes [12]. A CHD-free year may not be free of other CVDs like stroke, leading to an underestimation of the influence of genetic predisposition on CVD. Evaluating the overall CVD genetic risk is essential to understand its impact on LE free of any CVD and to what extent healthy lifestyles can offset this impact.

With the rapid emergence of large genome-wide association studies (GWAS), millions of genetic variants have been translated into polygenic risk scores (PRSs) for CVD, such as CAD and stroke, and their risk factors [13, 14]. Although there are some challenges in developing a PRS for overall CVD due to its heterogeneity, integrating genetic information of CVD subtypes and their risk factors to build a PRS is a promising strategy for characterizing the genetic risk of overall CVD. Similar approaches have been used to develop PRS for CAD [14, 15], stroke [16], overall cancer [17], and even all-cause mortality [18].

The current study first investigated the potential impact of genetic risk and lifestyles on the transition from CVD-free to CVD-onset and then death in the China Kadoorie Biobank (CKB). We then translated these complex relationships into more understandable metrics, specifically how genetic risk and lifestyle choices affected LE without CVD and total LE.

## Methods

### Study population

CKB is a large ongoing prospective cohort study. Details have been described elsewhere [19]. Briefly, CKB recruited 512,724 adults aged 30–79 from 5 rural and 5 urban areas across China during 2004–2008. In 2008 and 2013–2014, a random sample of about 5% of participants was surveyed twice. Trained staff administered a laptop-based questionnaire with built-in logic validation and missing detection for quality control and took physical measurements in the baseline and the resurveys. Blood samples were also collected for on-site blood glucose testing and long-term storage. The study was approved by the Ethics Committee of the Chinese Centre for Disease Control and Prevention (005/2004, Beijing, China), the Peking University Health Science Center (IRB00001052-20040, Beijing, China), and the Oxford Tropical Research Ethics Committee of the University of Oxford (025–04, Oxford, UK).

A total of 100,639 participants had complete baseline and genotyping data. The sampling process was detailed elsewhere [20]. Briefly, 75,981 participants were randomly selected from the entire cohort and the remaining 24,658 participants came from a case–control design nested in the cohort, including newly developed CVD and chronic obstructive pulmonary disease cases from baseline to January 1, 2014, as well as controls without these incident events. We excluded those who had self-reported heart disease or stroke diagnosed by a doctor at baseline. Of the remaining 96,400 participants, 24,251 of the case–control sample were used as a prospective training set for PRS construction. The other 72,149 participants of the random sample were used as a testing set for PRS evaluation and following prospective analysis.

### Assessment of lifestyles and covariates

By referring to previous studies and the American Heart Association’s Life’s Essential 8 [5, 7, 8, 21–23], we focused on six well-established lifestyle risk factors for CVD, including smoking, diet habits, physical activity, sleep duration, body mass index (BMI), and waist circumference. We defined current smoking or having stopped smoking due to illness as unfavorable, because both groups had a similarly elevated CVD risk [24]. Having any of the following diet habits was deemed unfavorable: not eating fresh vegetables or fruits on a daily basis, or eating red meat daily or less than weekly [22]. For physical activity, the unfavorable group included those who had total physical activity levels lower than the sex- and age-specific (< 50, 50–59, and ≥ 60 years) medians [25]. Sleeping less than 7 h or more than 9 h was defined as unfavorable [21]. Adiposity measures are usually used to reflect the overall energy balance and incorporated into the analysis of lifestyles. There is evidence that the association of central obesity with CVD is independent of BMI [26, 27]. Therefore, we considered unfavorable BMI (< 18.5 or ≥ 24.0 kg/m^2^) and WC (≥ 90 cm (men)/85 cm (women)) simultaneously. Assessments of lifestyles and socio-demographic characteristics were detailed in Supplementary methods (Additional file 1).

Lifestyles were categorized according to the numbers of unfavorable lifestyles as favorable (0–1), intermediate (2–4), and unfavorable (5–6). Among 24,109 participants attending the second resurvey, 71.0% kept their lifestyle risk categories consistent over an 8-year median follow-up period, regardless of CVD onset (Additional file 1: Table S1).

### Assessment of outcomes

Shortly after the baseline survey, disease incidence, hospitalization events, and death were obtained for all participants by linking to the local disease and death registries and the national health insurance database. More than 95% of the participants were successfully matched to the insurance database. We also conducted active follow-ups annually on participants who had failed links. The causes of disease and death were coded by trained staff using the 10 th revision of the International Classification of Disease (ICD-10). The primary outcome of interest was incident CVD (ICD-10: I00–I99). Three CVD subtypes, CAD (including fatal ischemic heart disease (I20–I25), nonfatal myocardial infarction (I21–I23), and coronary revascularization), ischemic stroke (IS, I63), and intracerebral hemorrhage (ICH, I61), were also included in the analysis. Case adjudication for ischemic heart disease and stroke is presented in the Supplementary methods (Additional file 1).

### Genotyping and construction of the MetaPRS

**Fig. 1:**
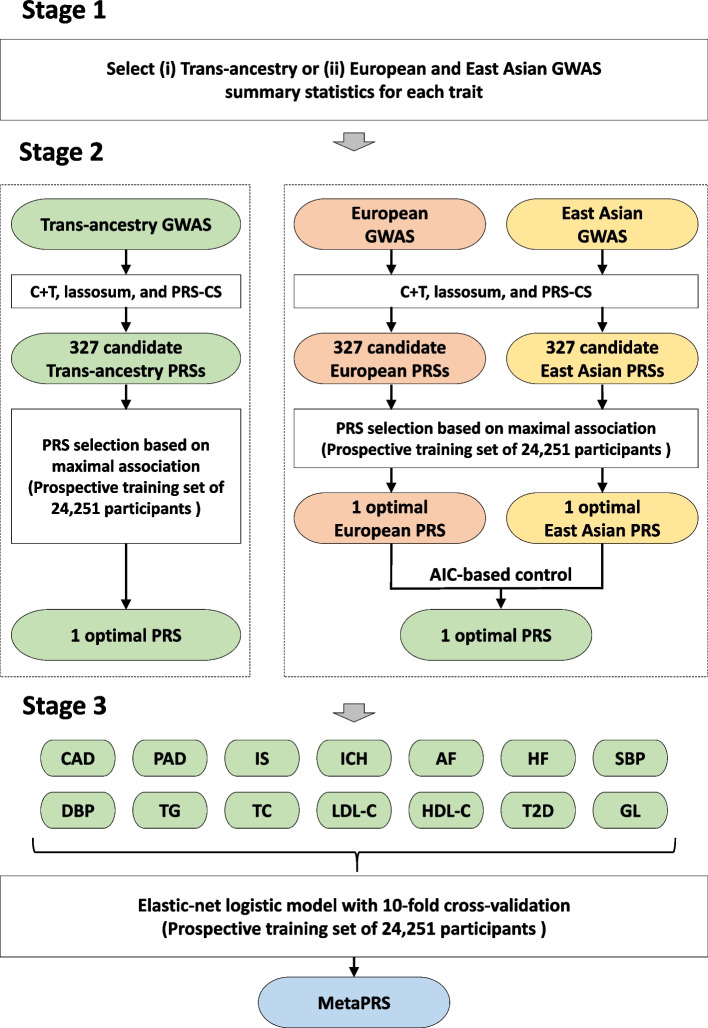
Flow chart of the MetaPRS construction. AF, atrial fibrillation; AIC, Akaike information criterion; CAD, coronary artery disease; CVD, cardiovascular disease; C + T, clumping and thresholding; DBP, diastolic blood pressure; GL, glucose level; GWAS, genome-wide association study; HDL-C, high-density lipoprotein cholesterol; HF, heart failure; ICH, intracerebral hemorrhage; IS, ischemic stroke; LDL-C, low-density lipoprotein cholesterol; PAD, peripheral arterial disease; PRS, polygenic risk score; SBP, systolic blood pressure; TC, total cholesterol; TG, triglycerides; T2D, type 2 diabetes

Genotyping was performed using two custom-designed arrays optimized for the Chinese population, each with 700 k and 803 k variants [20, 28]. Quality control and imputation were conducted centrally and detailed in Supplementary methods (Additional file 1). MetaPRSs for CVD and CVD subtypes were developed by combining different ancestry PRSs for CVD and CVD-related traits using a meta-scoring approach adapted from previous studies (Additional file 1: Table S2) [15, 16, 23, 29]. Briefly, the construction process comprised three stages (Fig. 1 and Additional file 1: Supplementary methods). First, we searched for the large-scale and publicly available GWAS summary statistics for 14 CVD and CVD-related risk factors across trans-ancestry, European, and East Asian populations. For each trait, if the trans-ancestry GWAS included the sample of the European and East Asian GWAS, it was selected. Otherwise, separate European and East Asian GWAS were used. Ultimately, we selected 7 trans-ancestry GWAS for 7 traits and 14 European and East Asian GWAS for another 7 traits (Additional file 1: Table S3) [30, 31, 13, 32–36]. Unified quality control was applied to align data quality before PRS construction (Additional file 1: Supplementary methods).

Second, we constructed 325 candidate PRSs using clumping and thresholding, lassosum [37], and PRS-CS [38] based on each GWAS. The optimal PRS for each GWAS was chosen based on its strongest association with the study outcomes, ensuring alignment with CVD risk. Most selected PRS were associated with CVD in the training set (Additional file 1: Table S4). For the European and East Asian PRS, we incorporated an Akaike information criterion (AIC)-based quality control procedure to integrate the genetic information from the two ancestries without being influenced by the heterogeneity and different genetic backgrounds. For example, for the systolic blood pressure PRS in European and East Asian ancestries, we determined whether to integrate the two or choose one of them based on their AIC when predicting the study outcome using a logistic model [14, 23]. This stage resulted in 14 correlated trait-specific PRSs (Additional file 1: Fig. S1).

Third, we used an elastic-net logistic model to integrate the 14 trait-specific PRSs with tenfold cross-validation to determine the penalized parameters. The low quality or highly correlated PRSs were penalized in the elastic-net model, and the MetaPRS consisting of 3,501,798 variants was constructed by combining the trait-specific PRSs using the elastic-net derived weights (Additional file 1: Fig. S2). We conducted sensitivity analyses to test the robustness of the construction process (Additional file 1: Supplementary methods). MetaPRS for CVD subtypes were constructed using the same pipeline but included different trait-specific PRSs (Additional file 1: Table S2).

Based on the MetaPRS quintiles, we classified participants’ disease-specific genetic risk as low (bottom quintile), intermediate (2nd–4 th quintile), or high (top quintile), referring to previous studies [5–7, 39, 40].

### Statistical analysis

We evaluated the association of the MetaPRS with incident CVD outcome in the testing set using a Cox proportional hazard model with age as the time scale. Person-time was calculated from age at study entry to age at CVD onset, death, loss to follow-up, or December 31, 2018, whichever came first. The model was adjusted for sex, highest education (no formal school or primary school, middle school, or high school or above), marital status (married or not married), top 10 genetic principal components, and genotyping arrays. The performance of the CVD MetaPRS was further assessed by comparing its association with CVD with that of trait-specific PRSs, previous CAD and IS PRSs, and MetaPRS for CVD subtypes.

In the testing set, we applied the population-based multistate life table (MSLT) to estimate the LE with and without CVD and its subtypes for different genetic and/or lifestyle risk groups in men and women separately. Details are described in our previous work [9]. Briefly, we defined three transitions: (1) from free of CVD to incident CVD, (2) from free of CVD to non-CVD death, and (3) from incident CVD to death. Only the first entry in a state was considered, and no backflow was allowed. First, we used Poisson regression to estimate the age-specific 1-year transition rate for each transition. Second, we calculated the 5-year age-specific prevalence of each genetic and/or lifestyle risk category in the population with and without incident CVD. Third, we used Cox models to estimate the hazard ratios (HRs) of the genetic and/or lifestyle risk categories with each transition. The models were adjusted for the same covariates in the prior MetaPRS model, but adjusted for study regions instead of genetic principal components and genotyping array in the model for lifestyles. Additionally, in the analysis of transition 3, the follow-up time after CVD onset was used as the time axis and the age at CVD onset was additionally adjusted. The proportional hazard assumption for genetic and/or lifestyle groups was tested using log–log plots of survival on a nominal scale, which revealed no violation.

The transition-specific probabilities of each category were derived from these results (Additional file 2) and finally used to construct the MSLTs. The MSLTs started at age 40 and ended at 90, as few participants were followed up outside this age range. The 95% CIs for LE were estimated with 10,000 runs of Monte Carlo simulation (parametric bootstrapping). The assumed distributions in the parametric bootstrapping were detailed in the Supplementary methods (Additional file 1).

Due to the high clustering of risk factors in CVD cases, collider bias may arise in the analysis of transition 3. We conducted a sensitivity analysis using the inverse probability weighting (IPW) approach to balance the distribution of risk factors between CVD cases and the total testing set [41], including the aforementioned covariates and prevalent hypertension and diabetes. Given the high prevalence of less healthy dietary habits in this study, we performed a sensitivity analysis to exclude diet habits and re-classified lifestyles according to the number of remaining unhealthy lifestyle factors into favorable (0–1), intermediate (2–3), and unfavorable (4–5). Additionally, we included the three diet habits as single lifestyle factors to re-classify lifestyles into favorable (0–3), intermediate (4–5), and unfavorable (6–8). We re-classified the genetic risk into three groups according to the tertiles of MetaPRS to test whether the results were sensitive to the classification. We also estimated the LE with and without CVD and its subtypes at age 50 and 65.

Bonferroni-corrected *p*-values were calculated by multiplying the original *p*-values with 18, which is the number of tests for the associations between the three main exposure variables (genetic and/or lifestyle groups) and three transitions in both sexes. Statistical significance was set at *p* < 0.05 with two sides test. All statistical analyses were performed using R version 4.0.3 for Linux.

## Results

### Study population

The mean ages (SD) of 24,251 and 72,149 participants in the training and testing sets were 58.2 (10.7) and 51.7 (10.5), respectively, with men accounting for 50.5% and 40.2% (Table 1 and Additional file 1: Table S5). Men had more unfavorable lifestyles and a significantly higher smoking prevalence in both sets than women.

**Table 1:** Baseline characteristics of the testing set

	All (*n* = 72,149)	Women (*n* = 43,170)	Men (*n* = 28,979)
Age, years ± SD	51.7 ± 10.5	51.2 ± 10.4	52.4 ± 10.8
Middle school and above, %	50.8	45.3	59.0
Married, %	90.8	89.2	93.3
Family history of CVD^a^, %	20.1	20.2	20.0
Prevalent hypertension, %	34.3	32.6	36.8
Prevalent diabetes, %	5.6	5.7	5.4
Having an unfavorable lifestyle^b^, %			
Current smoking	28.6	2.8	67.1
Less healthy dietary habits	92.7	91.0	95.1
Low physical activity	49.4	49.1	49.9
Unhealthy sleep duration	27.4	28.1	26.3
Unhealthy body mass index	48.8	50.2	46.8
Unhealthy waist circumference	22.1	23.3	20.4
Number of unfavorable lifestyle factors, %			
0–1 (favorable lifestyle)	15.5	21.4	6.8
2–4 (intermediate lifestyle)	77.6	74.9	81.6
5–6 (unfavorable lifestyle)	6.9	3.7	11.6

*SD *standard deviation, *CVD *cardiovascular disease

^a^Family history of CVD was defined as at least one of parents or siblings having heart disease or stroke

^b^Unhealthy lifestyles were defined as follows: current smoking or having stopped smoking because of illness; having any of the three dietary habits (not eating fresh fruits daily, not eating vegetables daily, or eating red meat daily or less than weekly); engaging in a sex- and age-specific lower half of total physical activity; sleeping < 7 or > 9 h/day; having BMI < 18.5 or ≥ 24.0 kg/m^2^; and having waist circumference ≥ 90 cm (men)/85 cm (women)

During 168,744 person-years (median 6.3 years) of follow-up in the training set, 17,183 participants developed CVD, including 3316 CAD, 6344 IS, and 5321 ICH. During 782,708 person-years (median 12.1 years) of follow-up in the testing set, 20,425 participants developed CVD, including 1745 CAD, 7506 IS, and 1193 ICH.

### Validation of the MetaPRS

In the testing set, the distribution of MetaPRS was approximately normally distributed, with a higher mean among CVD cases than noncases (Additional file 1: Fig. S3). The MetaPRS were associated with CVD risk, with an HR (95% CI) per SD increment of 1.20 (1.19–1.22), which was stronger than the associations of trait-specific PRSs, previous CAD and stroke PRS, and MetaPRS for CVD subtypes with CVD (Additional file 1: Fig. S4 and Table S6). The construction process was robust to heterogeneity, data quality variation, and genetic structural differences across GWAS, including rare variants and changing the training set, and was improved by integrating trans-ancestry information (Additional file 1: Figs. S5–S8 and Supplementary results).

### Associations of genetic risk and lifestyles with different transition stages

High genetic risk was associated with increased risks of incident CVD and death after CVD development, but the former was stronger than the latter (Table 2). The corresponding HRs (95% CIs) were 1.63 (1.54–1.73) and 1.25 (1.09–1.44) in women and 1.64 (1.53–1.76) and 1.14 (1.00–1.30) in men. The associations of unfavorable lifestyles with developing CVD and death after CVD were close, with HRs (95% CIs) of 1.49 (1.36–1.62) and 1.40 (1.13–1.73) in women and 1.80 (1.62–2.01) and 1.75 (1.39–2.20) in men, respectively (Table 2). We found no statistically significant associations of high genetic risk or unfavorable lifestyles with transitions from baseline to non-CVD death after Bonferroni correction. The effect sizes of genetic risk and lifestyles with death following CVD were basically unchanged in the sensitivity analysis of IPW (Additional file 1: Table S7). The risk of developing CVD increased with genetic risk and unhealthy lifestyle factors, with the highest HR of 2.30 (1.93–2.74) in women and 2.75 (2.16–3.51) in men for high genetic risk and unfavorable lifestyle group (Additional file 1: Tables S8–S9).

**Table 2:** Associations of genetic risk and lifestyles with different transition stages in the testing set

Sex	Category	Baseline → CVD			Baseline → non-CVD death			CVD → death		
		HR (95% CI)	*p*	Adjusted p^*^	HR (95% CI)	*p*	Adjusted p^*^	HR (95% CI)	*p*	Adjusted p^*^
Women	Genetic risk									
	Low	Reference			Reference			Reference		
	Intermediate	1.20 (1.15–1.26)	7.3E − 14	1.3E − 12	1.01 (0.87–1.16)	0.941	1	1.07 (0.95–1.21)	0.255	1
	High	1.63 (1.54–1.73)	1.2E − 65	2.2E − 64	1.11 (0.94–1.33)	0.224	1	1.25 (1.09–1.44)	0.002	0.028
	Lifestyles									
	Favorable	Reference			Reference			Reference		
	Intermediate	1.19 (1.13–1.26)	1.9E − 11	3.5E − 10	1.01 (0.87–1.18)	0.864	1	1.21 (1.05–1.40)	0.006	0.113
	Unfavorable	1.49 (1.36–1.62)	1.1E − 18	2E − 17	1.02 (0.76–1.37)	0.888	1	1.40 (1.13–1.73)	0.002	0.029
Men	Genetic risk									
	Low	Reference			Reference			Reference		
	Intermediate	1.23 (1.16–1.31)	2.3E − 12	4.1E − 11	1.02 (0.91–1.15)	0.699	1	1.01 (0.90–1.12)	0.878	1
	High	1.64 (1.53–1.76)	7.0E − 46	1.3E − 44	0.96 (0.82–1.12)	0.622	1	1.14 (1.00–1.29)	0.046	0.829
	Lifestyles									
	Favorable	Reference			Reference			Reference		
	Intermediate	1.26 (1.14–1.39)	3.2E − 06	5.7E − 05	1.36 (1.09–1.69)	0.005	0.098	1.47 (1.19–1.80)	1.3E − 04	0.002
	Unfavorable	1.80 (1.62–2.01)	3.4E − 26	6.1E − 25	1.33 (1.02–1.73)	0.032	0.578	1.75 (1.39–2.20)	7.5E − 07	1.4E − 05

*CVD *cardiovascular disease, *PY *person-year, *HR *hazard ratio, *CI *confidence interval

^*^Bonferroni-corrected *p*-values were calculated by multiplying the original *p*-values by 18

Hazard ratios and 95% confidence intervals were estimated with adjustment for highest education, marital status, and top 10 principal components and genotyping arrays in the analysis of genetic risk or study regions in the analysis of lifestyles

The genetic risk was categorized into low (bottom quintile), intermediate (2nd–4 th quintile), and high (top quintile) according to quintile cutoff points of the MetaPRS. The lifestyles were categorized into favorable (0–1), intermediate (2–4), and unfavorable (5–6) according to the number of unfavorable lifestyle factors

### Association of genetic risk and lifestyles with total and CVD-free LE

**Fig. 2:**
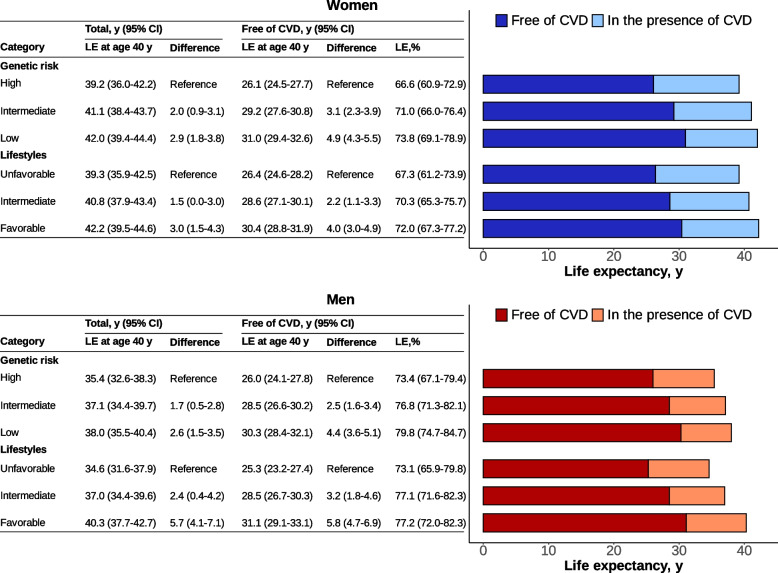
Life expectancy at age 40 with and without cardiovascular disease according to genetic risk and lifestyles in the testing set. CI, confidence interval; CVD, cardiovascular disease; LE, life expectancy. The genetic risk and lifestyles were categorized in the same way as in Table 2

Longer total LE and LE without CVD, as well as a higher proportion of LE without CVD to total LE at age 40, were linked to lower genetic risk and healthier lifestyles in both women and men (Fig. 2). In women, the increased LE without CVD associated with low genetic risk (4.9 years, 95% CI 4.3–5.5) was greater than the increased total LE (2.9, 1.8–3.8). Women with low genetic risk had a much higher proportion of LE without CVD than those with high genetic risk (73.8% vs. 66.6%). The extended LE without CVD associated with favorable lifestyle (4.0, 3.0–4.9) was slightly larger than the extended total LE (3.0, 1.5–4.3), resulting in a moderate difference in proportions of LE without CVD between favorable and unfavorable lifestyle categories (72.0% vs. 67.3%).

In men, the association patterns of low genetic risk with increased total LE, LE without CVD, and its proportion were similar to those in women. However, the difference in total LE (5.7, 4.1–7.1) and LE without CVD (5.8, 4.7–6.9) between lifestyle categories was very close. Participants with low genetic risk or favorable lifestyles also had a longer LE without CVD subtypes than those with high genetic risk or unfavorable lifestyles (Additional file 1: Fig. S9).

**Fig. 3:**
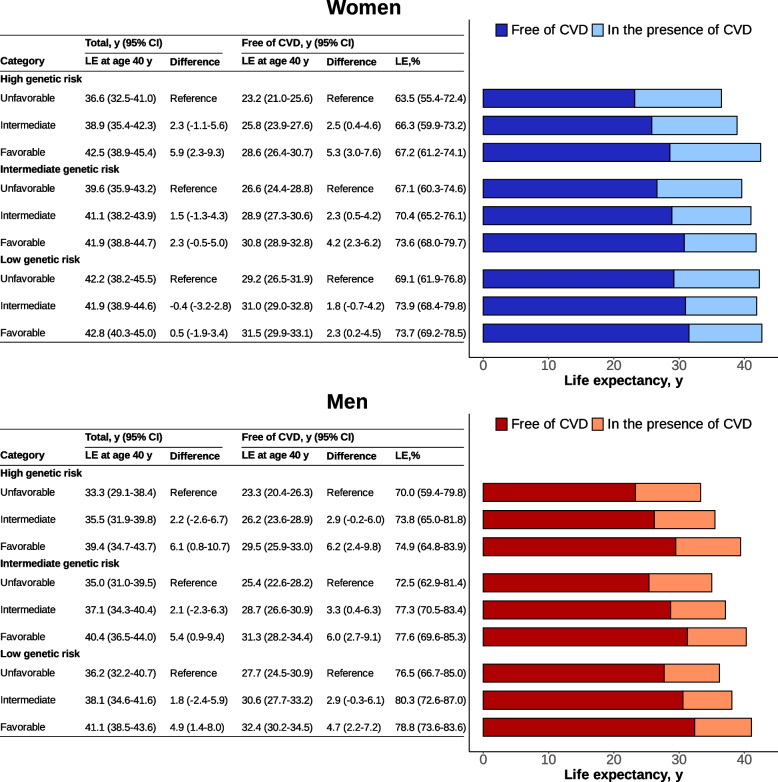
Life expectancy at age 40 with and without cardiovascular disease according to joint categories of genetic risk and lifestyles in the testing set. CI, confidence interval; CVD, cardiovascular disease; LE, life expectancy. The genetic risk and lifestyles were categorized in the same way as in Table 2

There were discernible gradients in total LE, LE without CVD, and its proportion across the joint genetic risk and lifestyle groups (Fig. 3 and Additional file 1: Table S10). Compared to those with high genetic risk and unfavorable lifestyles, participants with low genetic risk and favorable lifestyles had higher total LE and LE without CVD by 6.2 (3.1–9.0) and 8.3 (6.5–10.0) in women and 7.8 (3.8–11.1) and 9.1 (6.6–11.4) in men (Additional file 1: Table S10). Among those with high genetic risk, participants with favorable lifestyles had longer total LE and LE without CVD of 5.9 (2.3–9.3) and 5.3 (3.0–7.6) in women and 6.1 (0.8–10.7) and 6.2 (2.4–9.8) in men, respectively (Fig. 3). The increased total LE and LE without CVD associated with favorable lifestyles were smaller in participants with low and intermediate genetic risk than in those with high genetic risk, particularly in women. Sensitivity analyses—excluding dietary habits, incorporating specific dietary components as distinct lifestyle factors, and reclassifying genetic risk using tertiles of MetaPRS—demonstrated consistent patterns indicating that favorable lifestyles were associated with additional extensions in total and CVD-free LE among high genetic risk groups, despite some attenuation, with more pronounced benefits observed in women than in men (Additional file 1: Figs. S10–S12).

The associations of joint categories of genetic risk and lifestyles with LE without CVD subtypes were similar to the results of overall CVD (Additional file 1: Fig. S13). Within the same genetic risk group, changes in lifestyles from unfavorable to favorable were also associated with a longer LE, LE without CVD, and its proportion at age 50 or 65, though the differences were smaller than those at age 40 (Additional file 1: Figs. S14–S15).

## Discussion

In the current cohort study of Chinese adults, both genetic predispositions and lifestyles were associated with the risk of CVD incidence and death after CVD, but to varying extents. At age 40, low genetic risk was associated with a greater increase in CVD-free LE than the increase in total LE. In contrast, lifestyles had a comparable influence on CVD-free and total LE. Participants with high genetic risk gained significantly more CVD-free and total LE from favorable lifestyles, increasing by 5.3 and 5.9 years in women and 6.2 and 6.1 years in men, respectively.

In contrast to the large body of studies on CAD and stroke PRS, few studies systematically evaluated the ability of PRS to predict composite CVD outcomes [42–45], owing to limited genetic data for composite CVD outcomes and the heterogeneity of the CVD subtypes. Previous studies developed PRS for atherosclerotic CVD by incorporating either GWAS data or PRSs for CAD and stroke [42, 44]. However, these studies did not consider genetic predisposition for other CVD subtypes and CVD risk factors, which may limit the performance of genetic prediction for a broader CVD outcome (e.g., overall CVD), especially in the Chinese population with a high burden of ICH and other CVD subtypes [46]. In this study, we integrated six PRSs for CVD subtypes with the highest prevalence in China and eight PRSs for CVD risk factors to construct a MetaPRS for overall CVD. This MetaPRS had the strongest association with overall CVD when compared to trait-specific PRSs, previous CAD and stroke PRS, and other MetaPRSs tailored for CAD, IS, and ICH.

In the ARIC study of 8372 white and 2314 black individuals, with a median age of 54 years and a median follow-up of 26.4 years, the CHD-free and overall survival years of low genetic risk group (defined as having a CHD PRS < 20 th percentile) were 4.9 and 1.6 years longer than those of high genetic risk group (CHD PRS > 80 th percentile), respectively [6]. Similar results were obtained for white and black individuals, but with white individuals having a greater increase in CHD-free and overall survival years than black individuals. Our findings also revealed that in both men and women, the increase in LE without CVD associated with low genetic risk was greater than the increase in total LE. In other words, low genetic risk was associated with a longer CVD-free life span, freeing up space previously occupied by CVD and shortening the years of life lived with CVD. Our analyses contributed to a better understanding of this phenomenon. Total LE consists of LE without and with CVD; the former depends on the risk of CVD incidence and non-CVD death, and the latter is a result of mortality risk among participants with CVD [47]. In the current population, high genetic risk was strongly associated with CVD risk, but only weakly associated with death after CVD and not with non-CVD death.

Unlike the findings for genetic risk, this study found that unfavorable lifestyles were consistently associated with an increased risk of CVD incidence and mortality among those with CVD. Correspondingly, favorable lifestyles had a greater impact on LE without CVD than total LE, but the difference was smaller than the above-mentioned genetic findings, particularly among men. Previous studies also found that favorable lifestyles were linked to a similar extension of LE without CVD and total LE [9, 48, 49]. These findings suggested that maintaining favorable lifestyles resulted in significant benefits in LE without CVD and also survival after CVD onset, and thus substantially contributed to overall lifespan.

In the above ARIC study of white individuals with high genetic risk, an ideal LS7 score was associated with 20.2 and 12.4 years longer CHD-free and overall survival years, which showed greater benefits than those in the low genetic risk group, with corresponding increases of 11.4 and 8.4 years, respectively. However, an ideal LS7 score was associated with more CHD-free and overall survival years among black individuals with low genetic risk than those with high genetic risk. In a study of 39,755 Chinese adults with a mean follow-up of 12.9 years from the Prediction for Atherosclerotic Cardiovascular Disease Risk in China project, participants with high genetic risk had a greater benefit in CAD-free years associated with favorable cardiovascular health profiles than those with low genetic risk, extending by 2.6 and 1.1 years, respectively [50]. Our study expands on previous findings by providing broader estimates of LE without overall CVD or other subtypes, as well as total LE associated with genetic risk and lifestyles in a Chinese population. We found that adherence to favorable lifestyles can mitigate the negative effects of high genetic risk on both CVD-free and total LE in men and women. The beneficial impacts of favorable lifestyles were more pronounced in high genetic risk group than in low genetic risk group, particularly in women. One potential explanation for this sex difference is that genes and genetic pathways that contribute to high genetic risk may act differently in men and women, despite the MetaPRS being constructed in the same way for both sexes. For example, previous studies have demonstrated that APOE ε4 has a larger impact on women’s life years than on men’s life years [51, 52]. Furthermore, individuals carrying APOE ε4 are more sensitive to unhealthy diets, leading to an additional increase in LDL-C [53]. In other words, favorable lifestyles can avoid this additional harm that results from the interaction between high genetic risk and unfavorable lifestyles and provide greater benefits for women with high genetic risk.

In the current study, we used data from a geographically diverse Chinese population to first demonstrate the role of genetic risk and lifestyles on the relative risk of CVD onset and progression, and then translated these findings into their implications for LE without CVD and total LE for better communication and interpretation. This study has several strengths, including a large sample size of CKB participants, long-term follow-up, and a low rate of loss to follow-up. The continuous and dynamic collection of morbidity and mortality allows us to not only estimate total LE but also finely characterize all transition stages from free of CVD to developing CVD and death. We used a parsimonious and powerful approach to tailor a MetaPRS for predicting composite outcomes, namely overall CVD, and demonstrated its remarkable performance in overall CVD risk stratification. We trained and validated the MetaPRS in separate populations, following the guideline for PRS development and validation [54].

There are a few limitations to note. First, our current efforts are focused on LE free of CVD because CVD has relatively clear genetic determinants and well-established associations with lifestyles. However, our findings cannot be interpreted in terms of healthy LE, despite the fact that CVD is a major cause of mortality and morbidity across the world. Future research may include other important diseases. Second, lifestyles were assessed using self-report questionnaires. The measurement errors were inevitable but may not materially change the conclusion. Third, we only measured lifestyles for the whole population at baseline and did not account for the potential changes in lifestyles during the follow-up period. However, we found that for most participants in the 2013–2014 resurvey, the lifestyle risk category remained unchanged after a median follow-up of nearly 8 years, regardless of CVD occurrence during this period. Furthermore, the use of baseline lifestyles can avoid reverse causality. Finally, owing to the nature of the observational study, we cannot attribute causal interpretations to our findings.

## Conclusions

In this cohort study of Chinese adults, adopting healthy lifestyles could significantly mitigate the negative consequences of having a high genetic risk. Those with high genetic risk benefited more from improved lifestyles in extending CVD-free and total LE, particularly women. Even among men with low genetic risk, favorable lifestyles significantly improved CVD-free LE and total lifespan, but women with low genetic risk benefited primarily from delayed CVD onset. Our findings indicate that promoting healthy lifestyles throughout the population has important implications for healthy aging. With the growing popularity of genetic testing in the general population, communicating quantifiable benefits from adopting favorable lifestyles based on genetic risk may motivate lifestyle improvement and long-term persistence.

## Supplementary Information

Additional file 1. Supplementary material.docx: Supplementary methods. Supplementary results. Table S1. Changes in lifestyle risk categories by cardiovascular disease onset between 2004-08 baseline and 2013-14 resurvey. Table S2. Traits covered in the MetaPRSs for CVD and CVD subtypes. Table S3. Source of GWAS summary statistics used in the current study. Table S4. Associations of optimal PRSs for each GWAS (per standard deviation increment) with cardiovascular disease in the training set. Table S5. Baseline characteristics of the training set. Table S6. Associations of previous PRSs and disease-specific MetaPRSs with cardiovascular disease in the testing set. Table S7. Associations of genetic risk and lifestyles with transition 3 after inverse probability weighting in the testing set. Table S8. Associations of joint categories of genetic risk and lifestyles with different transitions in women in the testing set. Table S9. Associations of joint categories of genetic risk and lifestyles with different transitions in men in the testing set. Table S10. Differences in life expectancy at age 40 with and without cardiovascular disease between each joint category of genetic risk and lifestyles and the highest risk group in the testing set. Fig S1. Correlations among trait-specific PRSs in the training set. Fig S2. Associations of trait-specific PRSs (per standard deviation increment) with cardiovascular disease in the training set. Fig S3. Density plot of the MetaPRS in the testing set. Fig S4. Associations of the MetaPRS and trait-specific PRSs (per standard deviation increment) with cardiovascular disease in the testing set. Fig S5. Sensitivity analysis of building the MetaPRS using trans-ancestry GWAS summary statistics. Fig S6. Sensitivity analysis of building the MetaPRS including rare variants. Fig S7. Sensitivity analysis of building the MetaPRS using East Asian GWAS only. Fig S8. Sensitivity analysis of building the MetaPRS in the training set without COPD cases. Fig S9. Life expectancy at age 40 with and without cardiovascular disease subtypes according to genetic risk and lifestyles in the testing set. Fig S10. Life expectancy at age 40 with and without cardiovascular disease according to joint categories of genetic risk and lifestyles (excluding diet factors) in the testing set. Fig S11. Life expectancy at age 40 with and without cardiovascular disease according to joint categories of genetic risk and lifestyles (with specific diet habits as single lifestyle factors) in the testing set. Fig S12. Life expectancy at age 40 with and without cardiovascular disease according to joint categories of genetic risk (categorized by tertiles of the MetaPRS) and lifestyles in the testing set. Fig S13. Life expectancy at age 40 with and without cardiovascular disease subtypes according to joint categories of genetic risk and lifestyles in the testing set. Fig S14. Life expectancy at age 50 with and without cardiovascular disease according to joint categories of genetic risk and lifestyles in the testing set. Fig S15. Life expectancy at age 65 with and without cardiovascular disease according to joint categories of genetic risk and lifestyles in the testing set

Additional file 2. Input for the multistate lifetable.xlsx: Input 1. Coefficients in the Poisson regression. Input 2. Prevalence of the genetic and/or lifestyle groups. Input 3. Coefficients of the genetic and/or lifestyle groups in the Cox proportion hazard model

## Data Availability

The source data for the findings of this study are available as follows. Input data for the multistate lifetable are included in Additional File 2. Researchers can request access to non-genetic data of the China Kadoorie Biobank (CKB) through www.ckbiobank.org/site/Data+Access, with approval typically taking 1–2 months. Due to Chinese regulations on genetic data, genotype data cannot be deposited in a publicly accessible database to safeguard individuals'privacy. However, it can be shared upon reasonable request to the corresponding author, in compliance with China’s genetic data-sharing policies. The SNP weights of the MetaPRS are being deposited in the Genome Variation Map (https://ngdc.cncb.ac.cn/gvm/getProjectDetail?project=GVP000036) and the PGS Catalog (https://www.pgscatalog.org; publication ID: PGP000746, score ID: PGS005253). GWAS summary statistics supporting this study are available from the GWAS catalog (https://www.ebi.ac.uk/gwas/studies/GCST90132315 [55], https://www.ebi.ac.uk/gwas/studies/GCST90018890 [56], https://www.ebi.ac.uk/gwas/studies/GCST90104535 [57], https://www.ebi.ac.uk/gwas/studies/GCST90018870 [58], https://www.ebi.ac.uk/gwas/studies/GCST006061 [59], https://www.ebi.ac.uk/gwas/studies/GCST90162626 [60], https://www.ebi.ac.uk/gwas/studies/GCST006624 [61], https://www.ebi.ac.uk/gwas/studies/GCST90018752 [62], https://www.ebi.ac.uk/gwas/studies/GCST006630 [63], https://www.ebi.ac.uk/gwas/studies/GCST90018732 [64], https://www.ebi.ac.uk/gwas/studies/GCST90239664 [65], https://www.ebi.ac.uk/gwas/studies/GCST90018755 [66], https://www.ebi.ac.uk/gwas/studies/GCST90239676 [67], https://www.ebi.ac.uk/gwas/studies/GCST90018754 [68], https://www.ebi.ac.uk/gwas/studies/GCST90239658 [69], https://www.ebi.ac.uk/gwas/studies/GCST90018741 [70], https://www.ebi.ac.uk/gwas/studies/GCST90239652 [71], https://www.ebi.ac.uk/gwas/studies/GCST90018736 [72], https://www.ebi.ac.uk/gwas/studies/GCST90018955 [73]), the NBDC Human Database (https://humandbs.dbcls.jp/en/hum0014-v32 [74]), and the DIAGRAM consortium (http://diagram-consortium.org/downloads.html [75]). The 1000 genome project reference panels are available from the https://mathgen.stats.ox.ac.uk/impute/1000GP_Phase3.html.

## Abbreviations

CVD: Cardiovascular disease
CAD: Coronary artery disease
LE: Life expectancy
ARIC: Atherosclerosis Risk in Communities
LS7: Life’s Simple 7
CHD: Coronary heart disease
GWAS: Genome-wide association study
PRS: Polygenic risk score
CKB: China Kadoorie Biobank
BMI: Body mass index
ICD-10: 10Th revision of the International Classification of Disease
IS: Ischemic stroke
ICH: Intracerebral hemorrhage
AIC: Akaike information criterion
MSLT: Multistate lifetable
HR: Hazard ratio
CI: Confidence interval
IPW: Inverse probability weighting

## References

[1] Chen X, Giles J, Yao Y, Yip W, Meng Q, Berkman L, et al. The path to healthy ageing in China: a Peking University-Lancet Commission. Lancet (London, England). 2022;400(10367):1967–2006. 36423650 10.1016/S0140-6736(22)01546-X PMC9801271

[2] Collaborators GDaI. Global burden of 369 diseases and injuries in 204 countries and territories, 1990–2019: a systematic analysis for the Global Burden of Disease Study 2019. Lancet (London, England). 2020;396(10258):1204–22. 10.1016/S0140-6736(20)30925-9 PMC7567026 33069326

[3] Roth GA, Mensah GA, Johnson CO, Addolorato G, Ammirati E, Baddour LM, et al. Global Burden of Cardiovascular Diseases and Risk Factors, 1990–2019: Update From the GBD 2019 Study. J Am Coll Cardiol. 2020;76(25):2982–3021. 33309175 10.1016/j.jacc.2020.11.010 PMC7755038

[4] Arnett DK, Blumenthal RS, Albert MA, Buroker AB, Goldberger ZD, Hahn EJ, et al. 2019 ACC/AHA Guideline on the Primary Prevention of Cardiovascular Disease: A Report of the American College of Cardiology/American Heart Association Task Force on Clinical Practice Guidelines. Circulation. 2019;140(11):e596–646. 30879355 10.1161/CIR.0000000000000678 PMC7734661

[5] Khera AV, Emdin CA, Drake I, Natarajan P, Bick AG, Cook NR, et al. Genetic Risk, Adherence to a Healthy Lifestyle, and Coronary Disease. N Engl J Med. 2016;375(24):2349–58. 27959714 10.1056/NEJMoa1605086 PMC5338864

[6] Hasbani NR, Ligthart S, Brown MR, Heath AS, Bebo A, Ashley KE, et al. American Heart Association’s Life’s Simple 7: Lifestyle Recommendations, Polygenic Risk, and Lifetime Risk of Coronary Heart Disease. Circulation. 2022;145(11):808–18. 35094551 10.1161/CIRCULATIONAHA.121.053730 PMC8912968

[7] Said MA, Verweij N, van der Harst P. Associations of Combined Genetic and Lifestyle Risks With Incident Cardiovascular Disease and Diabetes in the UK Biobank Study. JAMA Cardiol. 2018;3(8):693–702. 29955826 10.1001/jamacardio.2018.1717 PMC6143077

[8] Rutten-Jacobs LC, Larsson SC, Malik R, Rannikmäe K, Sudlow CL, Dichgans M, et al. Genetic risk, incident stroke, and the benefits of adhering to a healthy lifestyle: cohort study of 306 473 UK Biobank participants. BMJ (Clinical research ed). 2018;363: k4168. 30355576 10.1136/bmj.k4168 PMC6199557

[9] China Kadoorie Biobank Collaborative G. Healthy lifestyle and life expectancy free of major chronic diseases at age 40 in China. Nat hum behav. 2023;7(9):1542–50. 10.1038/s41562-023-01624-7 PMC7615116 37430072

[10] Wilkins JT, Ning H, Berry J, Zhao L, Dyer AR, Lloyd-Jones DM. Lifetime risk and years lived free of total cardiovascular disease. JAMA. 2012;308(17):1795–801. 23117780 10.1001/jama.2012.14312 PMC3748966

[11] Global Cardiovascular Risk C, Magnussen C, Ojeda FM, Leong DP, Alegre-Diaz J, Amouyel P, et al. Global Effect of Modifiable Risk Factors on Cardiovascular Disease and Mortality. N Engl J Med. 2023;389(14):1273–85. 10.1056/NEJMoa2206916 PMC10589462 37632466

[12] Ntalla I, Kanoni S, Zeng L, Giannakopoulou O, Danesh J, Watkins H, et al. Genetic Risk Score for Coronary Disease Identifies Predispositions to Cardiovascular and Noncardiovascular Diseases. J Am Coll Cardiol. 2019;73(23):2932–42. 31196449 10.1016/j.jacc.2019.03.512

[13] Mishra A, Malik R, Hachiya T, Jürgenson T, Namba S, Posner DC, et al. Stroke genetics informs drug discovery and risk prediction across ancestries. Nature. 2022;611(7934):115–23. 36180795 10.1038/s41586-022-05165-3 PMC9524349

[14] Patel AP, Wang M, Ruan Y, Koyama S, Clarke SL, Yang X, et al. A multi-ancestry polygenic risk score improves risk prediction for coronary artery disease. Nat Med. 2023;29(7):1793–803. 37414900 10.1038/s41591-023-02429-x PMC10353935

[15] Lu X, Liu Z, Cui Q, Liu F, Li J, Niu X, et al. A polygenic risk score improves risk stratification of coronary artery disease: a large-scale prospective Chinese cohort study. Eur Heart J. 2022;43(18):1702–11. 35195259 10.1093/eurheartj/ehac093 PMC9076396

[16] Abraham G, Malik R, Yonova-Doing E, Salim A, Wang T, Danesh J, et al. Genomic risk score offers predictive performance comparable to clinical risk factors for ischaemic stroke. Nat Commun. 2019;10(1):5819. 31862893 10.1038/s41467-019-13848-1 PMC6925280

[17] Zhu M, Wang T, Huang Y, Zhao X, Ding Y, Zhu M, et al. Genetic Risk for Overall Cancer and the Benefit of Adherence to a Healthy Lifestyle. Cancer Res. 2021;81(17):4618–27. 34321244 10.1158/0008-5472.CAN-21-0836

[18] Meisner A, Kundu P, Zhang YD, Lan LV, Kim S, Ghandwani D, et al. Combined Utility of 25 Disease and Risk Factor Polygenic Risk Scores for Stratifying Risk of All-Cause Mortality. Am J Hum Genet. 2020;107(3):418–31. 32758451 10.1016/j.ajhg.2020.07.002 PMC7477009

[19] Chen Z, Chen J, Collins R, Guo Y, Peto R, Wu F, et al. China Kadoorie Biobank of 0.5 million people: survey methods, baseline characteristics and long-term follow-up. Int J Epidemiol. 2011;40(6):1652–66. 10.1093/ije/dyr120 PMC3235021 22158673

[20] Walters RG, Millwood IY, Lin K, Schmidt Valle D, McDonnell P, Hacker A, et al. Genotyping and population characteristics of the China Kadoorie Biobank. Cell Genom. 2023;3(8): 100361. 37601966 10.1016/j.xgen.2023.100361 PMC10435379

[21] Lloyd-Jones DM, Allen NB, Anderson CAM, Black T, Brewer LC, Foraker RE, et al. Life’s Essential 8: Updating and Enhancing the American Heart Association’s Construct of Cardiovascular Health: A Presidential Advisory From the American Heart Association. Circulation. 2022;146(5):e18–43. 35766027 10.1161/CIR.0000000000001078 PMC10503546

[22] Lv J, Yu C, Guo Y, Bian Z, Yang L, Chen Y, et al. Adherence to Healthy Lifestyle and Cardiovascular Diseases in the Chinese Population. J Am Coll Cardiol. 2017;69(9):1116–25. 28254173 10.1016/j.jacc.2016.11.076 PMC6675601

[23] Sun D, Ding Y, Yu C, Sun D, Pang Y, Pei P, et al. Joint impact of polygenic risk score and lifestyles on early- and late-onset cardiovascular diseases. Nat Hum Behav. 2024;8(9):1810–8. 38987358 10.1038/s41562-024-01923-7

[24] Chan KH, Wright N, Xiao D, Guo Y, Chen Y, Du H, et al. Tobacco smoking and risks of more than 470 diseases in China: a prospective cohort study. The Lancet Public health. 2022;7(12):e1014–26. 36462513 10.1016/S2468-2667(22)00227-4 PMC7613927

[25] Han YT, Hu YZ, Yu CQ, Guo Y, Pei P, Yang L, et al. Lifestyle, cardiometabolic disease, and multimorbidity in a prospective Chinese study. European Heart Journal. 2021;42(34):3374-+. 10.1093/eurheartj/ehab413 PMC8423468 34333624

[26] Dale CE, Fatemifar G, Palmer TM, White J, Prieto-Merino D, Zabaneh D, et al. Causal Associations of Adiposity and Body Fat Distribution With Coronary Heart Disease, Stroke Subtypes, and Type 2 Diabetes Mellitus: A Mendelian Randomization Analysis. Circulation. 2017;135(24):2373–88. 28500271 10.1161/CIRCULATIONAHA.116.026560 PMC5515354

[27] Chen GC, Arthur R, Iyengar NM, Kamensky V, Xue X, Wassertheil-Smoller S, et al. Association between regional body fat and cardiovascular disease risk among postmenopausal women with normal body mass index. Eur Heart J. 2019;40(34):2849–55. 31256194 10.1093/eurheartj/ehz391 PMC6933870

[28] Zhu Z, Li J, Si J, Ma B, Shi H, Lv J, et al. A large-scale genome-wide association analysis of lung function in the Chinese population identifies novel loci and highlights shared genetic aetiology with obesity. Eur Respir J. 2021;58(4):2100199. 33766948 10.1183/13993003.00199-2021 PMC8513692

[29] Lu X, Niu X, Shen C, Liu F, Liu Z, Huang K, et al. Development and Validation of a Polygenic Risk Score for Stroke in the Chinese Population. Neurology. 2021;97(6):e619–28. 34031205 10.1212/WNL.0000000000012263 PMC8424497

[30] Aragam KG, Jiang T, Goel A, Kanoni S, Wolford BN, Atri DS, et al. Discovery and systematic characterization of risk variants and genes for coronary artery disease in over a million participants. Nat Genet. 2022;54(12):1803–15. 36474045 10.1038/s41588-022-01233-6 PMC9729111

[31] Sakaue S, Kanai M, Tanigawa Y, Karjalainen J, Kurki M, Koshiba S, et al. A cross-population atlas of genetic associations for 220 human phenotypes. Nat Genet. 2021;53(10):1415–24. 34594039 10.1038/s41588-021-00931-x PMC12208603

[32] Roselli C, Chaffin MD, Weng LC, Aeschbacher S, Ahlberg G, Albert CM, et al. Multi-ethnic genome-wide association study for atrial fibrillation. Nat Genet. 2018;50(9):1225–33. 29892015 10.1038/s41588-018-0133-9 PMC6136836

[33] Levin MG, Tsao NL, Singhal P, Liu C, Vy HMT, Paranjpe I, et al. Genome-wide association and multi-trait analyses characterize the common genetic architecture of heart failure. Nat Commun. 2022;13(1):6914. 36376295 10.1038/s41467-022-34216-6 PMC9663424

[34] Evangelou E, Warren HR, Mosen-Ansorena D, Mifsud B, Pazoki R, Gao H, et al. Genetic analysis of over 1 million people identifies 535 new loci associated with blood pressure traits. Nat Genet. 2018;50(10):1412–25. 30224653 10.1038/s41588-018-0205-x PMC6284793

[35] Graham SE, Clarke SL, Wu KH, Kanoni S, Zajac GJM, Ramdas S, et al. The power of genetic diversity in genome-wide association studies of lipids. Nature. 2021;600(7890):675–9. 34887591 10.1038/s41586-021-04064-3 PMC8730582

[36] Mahajan A, Taliun D, Thurner M, Robertson NR, Torres JM, Rayner NW, et al. Fine-mapping type 2 diabetes loci to single-variant resolution using high-density imputation and islet-specific epigenome maps. Nat Genet. 2018;50(11):1505–13. 30297969 10.1038/s41588-018-0241-6 PMC6287706

[37] Mak TSH, Porsch RM, Choi SW, Zhou X, Sham PC. Polygenic scores via penalized regression on summary statistics. Genet Epidemiol. 2017;41(6):469–80. 28480976 10.1002/gepi.22050

[38] Ge T, Chen CY, Ni Y, Feng YA, Smoller JW. Polygenic prediction via Bayesian regression and continuous shrinkage priors. Nat Commun. 2019;10(1):1776. 30992449 10.1038/s41467-019-09718-5 PMC6467998

[39] Mega JL, Stitziel NO, Smith JG, Chasman DI, Caulfield M, Devlin JJ, et al. Genetic risk, coronary heart disease events, and the clinical benefit of statin therapy: an analysis of primary and secondary prevention trials. Lancet (London, England). 2015;385(9984):2264–71. 25748612 10.1016/S0140-6736(14)61730-X PMC4608367

[40] Lacaze P, Bakshi A, Riaz M, Polekhina G, Owen A, Bhatia HS, et al. Aspirin for Primary Prevention of Cardiovascular Events in Relation to Lipoprotein(a) Genotypes. J Am Coll Cardiol. 2022;80(14):1287–98. 36175048 10.1016/j.jacc.2022.07.027 PMC10025998

[41] Mitchell RE, Hartley AE, Walker VM, Gkatzionis A, Yarmolinsky J, Bell JA, et al. Strategies to investigate and mitigate collider bias in genetic and Mendelian randomisation studies of disease progression. PLoS Genet. 2023;19(2): e1010596. 36821633 10.1371/journal.pgen.1010596 PMC9949638

[42] Weale ME, Riveros-Mckay F, Selzam S, Seth P, Moore R, Tarran WA, et al. Validation of an Integrated Risk Tool, Including Polygenic Risk Score, for Atherosclerotic Cardiovascular Disease in Multiple Ethnicities and Ancestries. Am J Cardiol. 2021;148:157–64. 33675770 10.1016/j.amjcard.2021.02.032

[43] Sun L, Pennells L, Kaptoge S, Nelson CP, Ritchie SC, Abraham G, et al. Polygenic risk scores in cardiovascular risk prediction: A cohort study and modelling analyses. PLoS Med. 2021;18(1): e1003498. 33444330 10.1371/journal.pmed.1003498 PMC7808664

[44] Vassy JL, Posner DC, Ho YL, Gagnon DR, Galloway A, Tanukonda V, et al. Cardiovascular Disease Risk Assessment Using Traditional Risk Factors and Polygenic Risk Scores in the Million Veteran Program. JAMA Cardiol. 2023;8(6):564–74. 37133828 10.1001/jamacardio.2023.0857 PMC10157509

[45] Elliott J, Bodinier B, Bond TA, Chadeau-Hyam M, Evangelou E, Moons KGM, et al. Predictive Accuracy of a Polygenic Risk Score-Enhanced Prediction Model vs a Clinical Risk Score for Coronary Artery Disease. JAMA. 2020;323(7):636–45. 32068818 10.1001/jama.2019.22241 PMC7042853

[46] Ma Q, Li R, Wang L, Yin P, Wang Y, Yan C, et al. Temporal trend and attributable risk factors of stroke burden in China, 1990–2019: an analysis for the Global Burden of Disease Study 2019. The Lancet Public health. 2021;6(12):e897–906. 34838196 10.1016/S2468-2667(21)00228-0 PMC9047702

[47] Peeters A, Mamun AA, Willekens F, Bonneux L. A cardiovascular life history. A life course analysis of the original Framingham Heart Study cohort. European heart journal. 2002;23(6):458–66. 10.1053/euhj.2001.2838 11863348

[48] Li Y, Schoufour J, Wang DD, Dhana K, Pan A, Liu X, et al. Healthy lifestyle and life expectancy free of cancer, cardiovascular disease, and type 2 diabetes: prospective cohort study. BMJ (Clinical research ed). 2020;368: l6669. 31915124 10.1136/bmj.l6669 PMC7190036

[49] O’Doherty MG, Cairns K, O’Neill V, Lamrock F, Jorgensen T, Brenner H, et al. Effect of major lifestyle risk factors, independent and jointly, on life expectancy with and without cardiovascular disease: results from the Consortium on Health and Ageing Network of Cohorts in Europe and the United States (CHANCES). Eur J Epidemiol. 2016;31(5):455–68. 26781655 10.1007/s10654-015-0112-8 PMC4901087

[50] Cui Q, Liu Z, Li J, Liu F, Niu X, Shen C, et al. Impact of cardiovascular health and genetic risk on coronary artery disease in Chinese adults. Heart (British Cardiac Society). 2023;109(10):756–62. 36539268 10.1136/heartjnl-2022-321657

[51] Jukarainen S, Kiiskinen T, Kuitunen S, Havulinna AS, Karjalainen J, Cordioli M, et al. Genetic risk factors have a substantial impact on healthy life years. Nat Med. 2022;28(9):1893–901. 36097220 10.1038/s41591-022-01957-2 PMC9499866

[52] Joshi PK, Fischer K, Schraut KE, Campbell H, Esko T, Wilson JF. Variants near CHRNA3/5 and APOE have age- and sex-related effects on human lifespan. Nat Commun. 2016;7:11174. 27029810 10.1038/ncomms11174 PMC5438072

[53] Minihane AM, Jofre-Monseny L, Olano-Martin E, Rimbach G. ApoE genotype, cardiovascular risk and responsiveness to dietary fat manipulation. Proc Nutr Soc. 2007;66(2):183–97. 17466101 10.1017/S0029665107005435

[54] Choi SW, Mak TS, O’Reilly PF. Tutorial: a guide to performing polygenic risk score analyses. Nat Protoc. 2020;15(9):2759–72. 32709988 10.1038/s41596-020-0353-1 PMC7612115

[55] Aragam KG, Jiang T, Goel A, Kanoni S, Wolford BN, Atri DS, et al. Discovery and systematic characterization of risk variants and genes for coronary artery disease in over a million participants. GWAS Catalog. https://www.ebi.ac.uk/gwas/studies/GCST90132315. 2022. 10.1038/s41588-022-01233-6 PMC9729111 36474045

[56] Sakaue S, Kanai M, Tanigawa Y, Karjalainen J, Kurki M, Koshiba S, et al. A cross-population atlas of genetic associations for 220 human phenotypes. GWAS catalog. https://www.ebi.ac.uk/gwas/studies/GCST90018890. 2021. 10.1038/s41588-021-00931-x PMC12208603 34594039

[57] Mishra A, Malik R, Hachiya T, Jürgenson T, Namba S, Posner DC, et al. Stroke genetics informs drug discovery and risk prediction across ancestries. GWAS catalog. https://www.ebi.ac.uk/gwas/studies/GCST90104535. 2022. 10.1038/s41586-022-05165-3 PMC9524349 36180795

[58] Sakaue S, Kanai M, Tanigawa Y, Karjalainen J, Kurki M, Koshiba S, et al. A cross-population atlas of genetic associations for 220 human phenotypes. GWAS catalog. https://www.ebi.ac.uk/gwas/studies/GCST90018870. 2021. 10.1038/s41588-021-00931-x PMC12208603 34594039

[59] Roselli C, Chaffin MD, Weng LC, Aeschbacher S, Ahlberg G, Albert CM, et al. Multi-ethnic genome-wide association study for atrial fibrillation. GWAS catalog. https://www.ebi.ac.uk/gwas/studies/GCST006061. 2018. 10.1038/s41588-018-0133-9 PMC6136836 29892015

[60] Levin MG, Tsao NL, Singhal P, Liu C, Vy HMT, Paranjpe I, et al. Genome-wide association and multi-trait analyses characterize the common genetic architecture of heart failure. GWAS catalog. https://www.ebi.ac.uk/gwas/studies/GCST90162626. 2022. 10.1038/s41467-022-34216-6 PMC9663424 36376295

[61] Evangelou E, Warren HR, Mosen-Ansorena D, Mifsud B, Pazoki R, Gao H, et al. Genetic analysis of over 1 million people identifies 535 new loci associated with blood pressure traits. GWAS catalog. https://www.ebi.ac.uk/gwas/studies/GCST006624. 2018. 10.1038/s41588-018-0205-x PMC6284793 30224653

[62] Sakaue S, Kanai M, Tanigawa Y, Karjalainen J, Kurki M, Koshiba S, et al. A cross-population atlas of genetic associations for 220 human phenotypes. GWAS catalog. https://www.ebi.ac.uk/gwas/studies/GCST90018752. 2021. 10.1038/s41588-021-00931-x PMC12208603 34594039

[63] Evangelou E, Warren HR, Mosen-Ansorena D, Mifsud B, Pazoki R, Gao H, et al. Genetic analysis of over 1 million people identifies 535 new loci associated with blood pressure traits. GWAS catalog. https://www.ebi.ac.uk/gwas/studies/GCST006630. 2018. 10.1038/s41588-018-0205-x PMC6284793 30224653

[64] Sakaue S, Kanai M, Tanigawa Y, Karjalainen J, Kurki M, Koshiba S, et al. A cross-population atlas of genetic associations for 220 human phenotypes. GWAS catalog. https://www.ebi.ac.uk/gwas/studies/GCST90018732. 2021. 10.1038/s41588-021-00931-x PMC12208603 34594039

[65] Graham SE, Clarke SL, Wu KH, Kanoni S, Zajac GJM, Ramdas S, et al. The power of genetic diversity in genome-wide association studies of lipids. GWAS catalog. https://www.ebi.ac.uk/gwas/studies/GCST90239664. 2021. 10.1038/s41586-021-04064-3 PMC8730582 34887591

[66] Sakaue S, Kanai M, Tanigawa Y, Karjalainen J, Kurki M, Koshiba S, et al. A cross-population atlas of genetic associations for 220 human phenotypes. GWAS catalog. https://www.ebi.ac.uk/gwas/studies/GCST90018755. 2021. 10.1038/s41588-021-00931-x PMC12208603 34594039

[67] Graham SE, Clarke SL, Wu KH, Kanoni S, Zajac GJM, Ramdas S, et al. The power of genetic diversity in genome-wide association studies of lipids. GWAS catalog. https://www.ebi.ac.uk/gwas/studies/GCST90239676. 2021. 10.1038/s41586-021-04064-3 PMC8730582 34887591

[68] Sakaue S, Kanai M, Tanigawa Y, Karjalainen J, Kurki M, Koshiba S, et al. A cross-population atlas of genetic associations for 220 human phenotypes. GWAS catalog. https://www.ebi.ac.uk/gwas/studies/GCST90018754. 2021. 10.1038/s41588-021-00931-x PMC12208603 34594039

[69] Graham SE, Clarke SL, Wu KH, Kanoni S, Zajac GJM, Ramdas S, et al. The power of genetic diversity in genome-wide association studies of lipids. GWAS catalog. https://www.ebi.ac.uk/gwas/studies/GCST90239658 2021. 10.1038/s41586-021-04064-3 PMC8730582 34887591

[70] Sakaue S, Kanai M, Tanigawa Y, Karjalainen J, Kurki M, Koshiba S, et al. A cross-population atlas of genetic associations for 220 human phenotypes. GWAS catalog. https://www.ebi.ac.uk/gwas/studies/GCST90018741. 2021. 10.1038/s41588-021-00931-x PMC12208603 34594039

[71] Graham SE, Clarke SL, Wu KH, Kanoni S, Zajac GJM, Ramdas S, et al. The power of genetic diversity in genome-wide association studies of lipids. GWAS catalog. https://www.ebi.ac.uk/gwas/studies/GCST90239652. 2021. 10.1038/s41586-021-04064-3 PMC8730582 34887591

[72] Sakaue S, Kanai M, Tanigawa Y, Karjalainen J, Kurki M, Koshiba S, et al. A cross-population atlas of genetic associations for 220 human phenotypes. GWAS catalog. https://www.ebi.ac.uk/gwas/studies/GCST90018736. 2021. 10.1038/s41588-021-00931-x PMC12208603 34594039

[73] Sakaue S, Kanai M, Tanigawa Y, Karjalainen J, Kurki M, Koshiba S, et al. A cross-population atlas of genetic associations for 220 human phenotypes. GWAS catalog. https://www.ebi.ac.uk/gwas/studies/GCST90018955. 2021. 10.1038/s41588-021-00931-x PMC12208603 34594039

[74] Ishigaki K, Akiyama M, Kanai M, Takahashi A, Kawakami E, Sugishita H, et al. Large-scale genome-wide association study in a Japanese population identifies novel susceptibility loci across different diseases. NBDC Human Database. https://humandbs.dbcls.jp/en/hum0014-v32. 2020. 10.1038/s41588-020-0640-3 PMC7968075 32514122

[75] Mahajan A, Taliun D, Thurner M, Robertson NR, Torres JM, Rayner NW, et al. Fine-mapping type 2 diabetes loci to single-variant resolution using high-density imputation and islet-specific epigenome maps. DIAGRAM. http://diagram-consortium.org/downloads.html. 2018. 10.1038/s41588-018-0241-6 PMC6287706 30297969

