# Supplementary material for: Impact of genetic risk and lifestyles on cardiovascular disease-free and total life expectancy: a cohort study
Source: Genome Med. 2025 Jul 22;17:81. doi: 10.1186/s13073-025-01487-9 (PMC12285114; doi:10.1186/s13073-025-01487-9)
Supplement: Supplementary file 1 — Additional file 1. Supplementary material.docx: Supplementary methods. Supplementary results. Table S1. Changes in lifestyle risk categories by cardiovascular disease onset between 2004-08 baseline and 2013-14 resurvey. Table S2. Traits covered in the MetaPRSs for CVD and CVD subtypes. Table S3. Source of GWAS summary statistics used in the current study. Table S4. Associations of optimal PRSs for each GWAS (per standard deviation increment) with cardiovascular disease in the training set. Table S5. Baseline characteristics of the training set. Table S6. Associations of previous PRSs and disease-specific MetaPRSs with cardiovascular disease in the testing set. Table S7. Associations of genetic risk and lifestyles with transition 3 after inverse probability weighting in the testing set. Table S8. Associations of joint categories of genetic risk and lifestyles with different transitions in women in the testing set. Table S9. Associations of joint categories of genetic risk and lifestyles with different transitions in men in the testing set. Table S10. Differences in life expectancy at age 40 with and without cardiovascular disease between each joint category of genetic risk and lifestyles and the highest risk group in the testing set. Fig S1. Correlations among trait-specific PRSs in the training set. Fig S2. Associations of trait-specific PRSs (per standard deviation increment) with cardiovascular disease in the training set. Fig S3. Density plot of the MetaPRS in the testing set. Fig S4. Associations of the MetaPRS and trait-specific PRSs (per standard deviation increment) with cardiovascular disease in the testing set. Fig S5. Sensitivity analysis of building the MetaPRS using trans-ancestry GWAS summary statistics. Fig S6. Sensitivity analysis of building the MetaPRS including rare variants. Fig S7. Sensitivity analysis of building the MetaPRS using East Asian GWAS only. Fig S8. Sensitivity analysis of building the MetaPRS in the training set without COPD cases. F [file 13073_2025_1487_MOESM1_ESM.docx]

**Supplementary material**

**[Supplementary methods](#_Toc192236981)** [3](#_Toc192236981)

[**Members of the China Kadoorie Biobank collaborative group** 3](#_Toc192236982)

[**Assessment and definition of lifestyles** 4](#_Toc192236983)

[**Cases adjudication** 4](#_Toc192236984)

[**Quality control in genotyping and imputation** 5](#_Toc192236985)

[**Construction of the MetaPRS** 5](#_Toc192236986)

[**Sensitivity analysis of the MetaPRS construction** 6](#_Toc192236987)

[**Distribution assumption in the parametric bootstrap** 7](#_Toc192236988)

[**Supplementary results** 7](#_Toc192236989)

[**Results of sensitivity analysis** 7](#_Toc192236990)

[**Table S1. Changes in lifestyle risk categories by cardiovascular disease onset between 2004-08 baseline and 2013-14 resurvey** 8](#_Toc192236991)

[**Table S2. Traits covered in the MetaPRSs for CVD and CVD subtypes** 9](#_Toc192236992)

[**Table S3. Source of GWAS summary statistics used in the current study** 10](#_Toc192236993)

[**Table S4. Associations of optimal PRSs for each GWAS (per standard deviation increment) with cardiovascular disease in the training set** 11](#_Toc192236994)

[**Table S5. Baseline characteristics of the training set** 13](#_Toc192236995)

[**Table S6. Associations of previous PRSs and disease-specific MetaPRSs with cardiovascular disease in the testing set** 14](#_Toc192236996)

[**Table S7. Associations of genetic risk and lifestyles with transition 3 after inverse probability weighting in the testing set** 15](#_Toc192236997)

[**Table S8. Associations of joint categories of genetic risk and lifestyles with different transitions in women in the testing set** 16](#_Toc192236998)

[**Table S9. Associations of joint categories of genetic risk and lifestyles with different transitions in men in the testing set** 17](#_Toc192236999)

[**Table S10. Differences in life expectancy at age 40 with and without cardiovascular disease between each joint category of genetic risk and lifestyles and the highest risk group in the testing set** 18](#_Toc192237000)

[**Fig S1. Correlations among trait-specific PRSs in the training set** 19](#_Toc192237001)

[**Fig S2. Associations of trait-specific PRSs (per standard deviation increment) with cardiovascular disease in the training set** 20](#_Toc192237002)

[**Fig S3. Density plot of the MetaPRS in the testing set** 21](#_Toc192237003)

[**Fig S4. Associations of the MetaPRS and trait-specific PRSs (per standard deviation increment) with cardiovascular disease in the testing set** 22](#_Toc192237004)

[**Fig S5. Sensitivity analysis of building the MetaPRS using trans-ancestry GWAS summary statistics** 23](#_Toc192237005)

[**Fig S6. Sensitivity analysis of building the MetaPRS including rare variants** 24](#_Toc192237006)

[**Fig S7. Sensitivity analysis of building the MetaPRS using East Asian GWAS only** 25](#_Toc192237007)

[**Fig S8. Sensitivity analysis of building the MetaPRS in the training set without COPD cases** 26](#_Toc192237008)

[**Fig S9. Life expectancy at age 40 with and without cardiovascular disease subtypes according to genetic risk and lifestyles in the testing set** 28](#_Toc192237009)

[**Fig S10. Life expectancy at age 40 with and without cardiovascular disease according to joint categories of genetic risk and lifestyles (excluding diet factors) in the testing set** 29](#_Toc192237010)

[**Fig S11. Life expectancy at age 40 with and without cardiovascular disease according to joint categories of genetic risk and lifestyles (with specific diet habits as single lifestyle factors) in the testing set** 30](#_Toc192237011)

[**Fig S12. Life expectancy at age 40 with and without cardiovascular disease according to joint categories of genetic risk (categorized by tertiles of the MetaPRS) and lifestyles in the testing set** 31](#_Toc192237012)

[**Fig S13. Life expectancy at age 40 with and without cardiovascular disease subtypes according to joint categories of genetic risk and lifestyles in the testing set** 33](#_Toc192237013)

[**Fig S14. Life expectancy at age 50 with and without cardiovascular disease according to joint categories of genetic risk and lifestyles in the testing set** 34](#_Toc192237014)

[**Fig S15. Life expectancy at age 65 with and without cardiovascular disease according to joint categories of genetic risk and lifestyles in the testing set** 35](#_Toc192237015)

[**Reference** 36](#_Toc192237016)

**Supplementary** **methods**

**Members of the China Kadoorie Biobank collaborative group**

**International Steering Committee:** Junshi Chen, Zhengming Chen (PI), Robert Clarke, Rory Collins, Liming Li (PI), Jun Lv, Richard Peto, Robin Walters.

**International Co-ordinating Centre, Oxford:** Daniel Avery, Maxim Barnard, Derrick Bennett, Lazaros Belbasis, Ruth Boxall, Ka Hung Chan, Yiping Chen, Zhengming Chen, Charlotte Clarke, Johnathan Clarke; Robert Clarke, Huaidong Du, Ahmed Edris Mohamed, Hannah Fry, Simon Gilbert, Pek Kei Im, Andri Iona, Maria Kakkoura, Christiana Kartsonaki, Hubert Lam, Kuang Lin, James Liu, Mohsen Mazidi, Iona Millwood, Sam Morris, Qunhua Nie, Alfred Pozarickij, Maryanm Rahmati, Paul Ryder, Saredo Said, Dan Schmidt, Becky Stevens, Iain Turnbull, Robin Walters, Baihan Wang, Lin Wang, Neil Wright, Ling Yang, Xiaoming Yang, Pang Yao.

**National Co-ordinating Centre, Beijing:** Xiao Han, Can Hou, Qingmei Xia, Chao Liu, Jun Lv, Pei Pei, Dianjianyi Sun, Canqing Yu, Lang Pan.

**10 Regional Co-ordinating Centres: Qingdao CDC:** Zengchang Pang, Ruqin Gao, Shanpeng Li, Haiping Duan, Shaojie Wang, Yongmei Liu, Ranran Du, Yajing Zang, Liang Cheng, Xiaocao Tian, Hua Zhang, Yaoming Zhai, Feng Ning, Xiaohui Sun, Feifei Li. **Licang CDC:** Silu Lv, Junzheng Wang, Wei Hou. **Heilongjiang Provincial CDC:** Wei Sun, Shichun Yan, Xiaoming Cui. **Nangang CDC:** Chi Wang, Zhenyuan Wu,Yanjie Li, Quan Kang. **Hainan Provincial CDC:** Huiming Luo, Tingting Ou. **Meilan CDC:** Xiangyang Zheng, Zhendong Guo, Shukuan Wu, Yilei Li, Huimei Li. **Jiangsu Provincial CDC:** Ming Wu, Yonglin Zhou, Jinyi Zhou, Ran Tao, Jie Yang, Jian Su. **Suzhou CDC:** Fang Liu, Jun Zhang, Yihe Hu, Yan Lu, Liangcai Ma, Aiyu Tang, Shuo Zhang, Jianrong Jin, Jingchao Liu. **Guangxi Provincial CDC:** Mei Lin, Zhenzhen Lu. **Liuzhou CDC:** Lifang Zhou, Changping Xie, Jian Lan,Tingping Zhu,Yun Liu, Liuping Wei, Liyuan Zhou, Ningyu Chen, Yulu Qin, Sisi Wang. **Sichuan Provincial CDC:** Xianping Wu, Ningmei Zhang, Xiaofang Chen, Xiaoyu Chang. **Pengzhou CDC:** Mingqiang Yuan, Xia Wu, Xiaofang Chen, Wei Jiang, Jiaqiu Liu, Qiang Sun. **Gansu Provincial CDC:** Faqing Chen, Xiaolan Ren, Caixia Dong. **Maiji CDC:** Hui Zhang, Enke Mao, Xiaoping Wang, Tao Wang, Xi zhang. **Henan Provincial CDC:** Kai Kang, Shixian Feng, Huizi Tian, Lei Fan. **Huixian CDC:** XiaoLin Li, Huarong Sun, Pan He, Xukui Zhang. **Zhejiang Provincial CDC:** Min Yu, Ruying Hu, Hao Wang. **Tongxiang CDC**: Xiaoyi Zhang, Yuan Cao, Kaixu Xie, Lingli Chen, Dun Shen. **Hunan Provincial CDC:** Xiaojun Li, Donghui Jin, Li Yin, Huilin Liu, Zhongxi Fu. **Liuyang CDC:** Xin Xu, Hao Zhang, Jianwei Chen,Yuan Peng, Libo Zhang, Chan Qu.

**Assessment and definition of lifestyles**

Lifestyles and demographical characteristics were collected using self-reported questionnaires and physical measurements. For ever smokers, we gathered information on the frequency, type, and daily amount of tobacco consumed. Former smokers were asked about the duration since quitting and the reasons for quitting. We assessed dietary patterns by collecting data on habitual intakes of 12 conventional food groups over the past 12 months using a validated qualitative food frequency questionnaire(1). To assess physical activity, we posed questions regarding the usual type and duration of occupational, commuting, domestic, and leisure-time activities within the past 12 months. We then calculated total physical activity levels in terms of metabolic equivalent task-hours per day (MET-h/d) by multiplying the hours spent on each activity by its corresponding physical intensity (MET) and summing up the MET-h/d for all activities. For sleeping, we asked about average daily sleep duration (including naps). Trained staff measured participants’ height, weight, and waist circumference using calibrated instruments. The body mass index (BMI) was subsequently calculated by dividing weight (kg) by the square of standing height (m).

**Cases adjudication**

To adjudicate ischemic heart disease (IHD) and stroke, CKB staff retrieved and photographed medical records of all reported cases from available hospitals using portable devices with high-resolution cameras(2). Cardiovascular experts examined these records to verify and determine the diagnoses. By December 2018, medical records of 30,974 IHD cases and 39,319 stroke cases without CVD history at baseline were retrieved and reviewed. The diagnosis was confirmed for 93.1% of IHD cases, 91.2% of ischemic stroke cases, and 92.5% of intracerebral hemorrhage cases.

**Quality control in genotyping and imputation**

After genotyping, we excluded variants with call rate<0.98, plate or batch effect *p*<10^−6^, Hardy–Weinberg equilibrium (HWE) deviations *p*<10^−6^, or minor allele frequency (MAF) difference from 1000 Genomes Project (1KGP) East Asian frequencies>0.2. Subsequently, imputation was performed using IMPUTE 4 based on haplotypes derived from the 1KGP Phase 3.

**Construction of the MetaPRS**

PRS is a quantitative metric for genetic predisposition derived from a weighted sum of the common variants of the genome. The weights are typically assigned based on the adjusted or unadjusted effect sizes from GWAS. Three analytic stages were conducted in the training set to generate the MetaPRS.

First, we identified two types of traits: (Ⅰ) six CVD subtypes with the highest prevalence in the Chinese population(3) and genetic factors contributing to at least some of their risk(4); and (Ⅱ) eight well-established risk factors for CVD, except lifestyles, which were exposure of interest in the study (Table S2). Then, we searched for large-scale and publicly available GWAS summary statistics across trans-ancestry, European, and East Asian population for each trait. If the searched trans-ancestry GWAS covered the population in the European and East Asian GWAS, we used the trans-ancestry GWAS to build PRS, if not, the European and East Asian GWAS were used (Table S3). None of the chosen GWAS overlapped with CKB. Before PRS construction, we excluded SNPs on sex chromosomes or autosomal SNPs with MAF<1% in either CKB or the GWAS original population, INFO<0.8, or ambiguous strands (A/T or C/G).

Second, three algorithms were implemented for PRS construction: clumping and thresholding (C+T), lassosum, and PRS-CS. C+T uses a 'clumping' approach to account for linkage disequilibrium (LD) and selects SNPs associated with the target trait at predetermined *p*-values. LD r^2^ (0, 0.2, 0.4, 0.6, 0.8, 1) and *p*-value thresholding (40 values from 5×10^-8^ to 1) must be tuned. Lassosum uses a penalized regression framework to adjust the effect sizes of SNPs (5). We followed the developer's recommendations and set a range of s (0.2, 0.5, 0.9, 1) and lambda (20 values from 0.001 to 0.1). PRS-CS employs a high-dimensional Bayesian framework to apply a continuous shrinkage on prior SNP effect sizes from GWAS(6). We used the software to search for the parameter phi automatically and set a range of phi (1×10^-6^, 1×10^-4^, 1×10^-2^, 1) for grid search. The 1KGP East Asian were extracted to serve as the reference panel for PRS derived from East Asian GWAS; otherwise, the 1KGP European were used. The associations of PRSs with incident CVD were estimated using Cox proportional hazard model with age as the time axis, stratified by sex, and adjustments for the top 10 genetic principal components (PCs) and genotyping arrays. We chose the PRS with the largest effect size as the best PRS for each GWAS. For SBP, DBP, TC, TG, LDL-C, HDL-C, and T2D, we constructed PRSs based on European and East Asian GWAS, respectively, and for each of the 7 traits, we determined whether to integrate the European and East Asian PRS or choose one of them based on their AIC when predicting the study outcome using a logistic model(7, 8).

Third, the elastic-net logistic models with different penalized parameters were implemented to estimate a weight for each standardized trait-specific PRS in order to account for the correlation between these components. Using 10-fold cross-validation, we chose the model with the highest area under the receiver operating characteristic curve in predicting CVD. The trait-specific PRSs were then linearly combined into a MetaPRS according to their weights.

We constructed MetaPRSs for the three CVD subtypes using a similar strategy, considering different trait-specific PRSs to integrate.

**Sensitivity analysis of the MetaPRS construction**

Heterogeneity, variation in GWAS quality, and genetic structural differences between European and East Asian population may interfere the MetaPRS's performance. To assess whether the AIC-based control procedure accounted for these interference, we build the trait-specific PRSs using summary statistics from the meta-analysis of the European and East Asian GWAS with METAL(9), to skip the AIC-based control procedure. Additionally, we used only the East Asian GWAS to build the MetaPRS(10) (11) (12) (13) (14).

To evaluate the value of rare variants (MAF<1%), we performed a sensitivity analysis by building the PRS with inclusion of rare variants that were either directly genotyped using arrays or imputed with an INFO>0.9.

We exclude the incident COPD cases in the training set and reconstructed the MetaPRS, to assess whether the enriched COPD cases in the training set distort the construction process.

**Distribution assumption in the parametric bootstrap**

The inputs for the multistate lifetables included (Ⅰ) the coefficients of age in the Poisson regression, which were assumed to follow a normal distribution, (Ⅱ) the prevalence of genetic and/or lifestyle groups, which were assumed to follow a normal distribution, (Ⅲ) the HRs of genetic and/or lifestyle groups with each transition, which were assumed to follow a lognormal distribution. These distributions were assumed according to previous studies(15-18).

**Supplementary results**

**Results of sensitivity analysis**

Sensitivity analysis of skipping the AIC-based control procedure led to a decreased association of the MetaPRS with CVD, suggesting that the AIC-based control procedure effectively accounts for heterogeneity and variation in data quality (Fig S5). Including rare variants did not alter the MetaPRS's performance obviously (Fig S6). Using only the East Asian GWAS yielded a MetaPRS with limited performance, indicating the improvement of integrating trans-ancestry information (Fig S7). The association of MetaPRS did not changed significantly when excluding the incident COPD cases in the training set (Fig S8).

**Table S1. Changes in lifestyle risk categories by cardiovascular disease onset between 2004-08 baseline and 2013-14 resurvey**

|  | No CVD onset  between the two surveys | CVD onset  between the two surveys | Total |
| --- | --- | --- | --- |
| Stable | 14,375 (71.0) | 2,744 (71.0) | 17,119 (71.0) |
| Worse | 3,595 (17.8) | 730 (18.9) | 4,325 (17.9) |
| Better | 2,273 (11.2) | 392 (10.1) | 2,665 (11.1) |

CVD, cardiovascular disease.

Numbers (percentages) were presented. Lifestyles were categorized in the same way as in Table 2. Changes in lifestyle risk were classified into stable (at the same risk level), worse (from low risk to high risk level), and better (from high risk to low risk level).

**Table S2. Traits covered in the MetaPRSs for CVD and CVD subtypes**

| Trait | Disease-specific MetaPRS | | | |
| --- | --- | --- | --- | --- |
|  | CVD | CAD | IS | ICH |
| CAD | √ | √ | √ | √ |
| Peripheral arterial disease | √ |  |  |  |
| IS | √ | √ | √ | √ |
| ICH | √ |  |  | √ |
| Atrial fibrillation | √ |  | √ |  |
| Heart failure | √ |  |  |  |
| Systolic blood pressure | √ | √ | √ | √ |
| Diastolic blood pressure | √ | √ | √ | √ |
| Triglycerides | √ | √ | √ |  |
| Total cholesterol | √ | √ | √ |  |
| Low-density lipoprotein cholesterol | √ | √ | √ |  |
| High-density lipoprotein cholesterol | √ | √ | √ |  |
| Type 2 diabetes | √ | √ | √ | √ |
| Glucose level | √ | √ | √ | √ |

CAD, coronary artery disease; IS, ischemic stroke; ICH, intracerebral hemorrhage; CVD, cardiovascular disease.

**Table S3. Source of GWAS summary statistics used in the current study**

| Trait | Ancestry | Sample size (case/noncase) | GWAS catalog ID | Reference |
| --- | --- | --- | --- | --- |
|  |  |  |  |  |
| Coronary artery disease | European/East Asian | 210,842/1,167,328 | GCST90132315 | Aragam et al.(19) |
| Peripheral arterial disease | European/East Asian | 11,226/649,565 | GCST90018890 | Sakaue et al.(11) |
| Ischemic Stroke | European/East Asian/South Asian/African American/Hispanic | 86,668/1,503,898 | GCST90104535 | Mishra et al.(20) |
| Intracerebral hemorrhage | European/East Asian | 3,391/623,600 | GCST90018870 | Sakaue et al.(11) |
| Atrial fibrillation | European/Japanese/African American/Hispanic | 65,446/522,744 | GCST006061 | Roselli et al.(21) |
| Heart failure | European/East Asian/African | 115,150/1,550,331 | GCST90162626 | Levin et al.(22) |
| Systolic blood pressure | European | 757,601 | GCST006624 | Evangelou et al.(23) |
|  | East Asian | 145,505 | GCST90018752 | Sakaue et al.(11) |
| Diastolic blood pressure | European | 757,601 | GCST006630 | Evangelou et al.(23) |
|  | East Asian | 145,515 | GCST90018732 | Sakaue et al.(11) |
| Triglycerides | European | 1,320,016 | GCST90239664 | Graham et al.(24) |
|  | East Asian | 111,667 | GCST90018755 | Sakaue et al.(11) |
| Total cholesterol | European | 1,320,016 | GCST90239676 | Graham et al.(24) |
|  | East Asian | 135,808 | GCST90018754 | Sakaue et al.(11) |
| Low density lipoprotein cholesterol | European | 1,320,016 | GCST90239658 | Graham et al.(24) |
|  | Japanese | 72,866 | GCST90018741 | Sakaue et al.(11) |
| High density lipoprotein cholesterol | European | 1,320,016 | GCST90239652 | Graham et al.(24) |
|  | East Asian | 74,970 | GCST90018736 | Sakaue et al.(11) |
| Type 2 diabetes | European | 74,124/824,006 | GCST009379 | Mahajan et al.(25) |
|  | Japanese | 40,250/170,615 | GCST90013693 | Ishigaki et al.(13) |
| Glucose level | European/East Asian | 448,252 | GCST90018955 | Sakaue et al.(11) |

**Table S4. Associations of optimal PRSs for each GWAS (per standard deviation increment) with cardiovascular disease in the training set**

| Trait | Ancestry | Method | Parameter 1^*^ | Parameter 2^†^ | No. of variants | HR (95% CI) |
| --- | --- | --- | --- | --- | --- | --- |
| Coronary artery disease | Trans-ancestry | C+T | 0.8 | 0.2 | 270,304 | 1.07 (1.05-1.08) |
| Peripheral arterial disease | Trans-ancestry | PRS-CS | 1.00E-04 | NA | 965,261 | 1.05 (1.03-1.06) |
| Ischemic stroke | Trans-ancestry | lassosum | 0.9 | 0.0020691 | 49,422 | 1.09 (1.07-1.10) |
| Intracerebral hemorrhage | Trans-ancestry | PRS-CS | auto | NA | 965,252 | 1.05 (1.03-1.06) |
| Atrial fibrillation | Trans-ancestry | lassosum | 1 | 0.001274275 | 310,539 | 1.04 (1.03-1.06) |
| Heart failure | Trans-ancestry | C+T | 0.6 | 1 | 617,003 | 1.11 (1.09-1.12) |
| Systolic blood pressure | European | PRS-CS | auto | NA | 954,691 | 1.12 (1.11-1.14) |
|  | East Asian | PRS-CS | 1.00E-04 | NA | 965,670 | 1.09 (1.08-1.11) |
| Diastolic blood pressure | European | lassosum | 0.9 | 0.001274275 | 236,291 | 1.13 (1.11-1.14) |
|  | East Asian | PRS-CS | 1.00E-04 | NA | 965,667 | 1.07 (1.06-1.09) |
| Triglycerides | European | C+T | 0 | 0.009 | 5,652 | 1.03 (1.02-1.05) |
|  | East Asian | lassosum | 0.5 | 0.004281332 | 144,266 | 1.02 (1.00-1.03) |
| Total cholesterol | European | lassosum | 0.2 | 0.0026367 | 30,698 | 1.02 (1.01-1.04) |
|  | East Asian | C+T | 0 | 5.00E-08 | 53 | 1.03 (1.01-1.04) |
| Low-density lipoprotein cholesterol | European | lassosum | 0.2 | 0.0026367 | 26,297 | 1.02 (1.00-1.03) |
|  | East Asian | lassosum | 0.2 | 0.023357215 | 147 | 1.01 (1.00-1.03) |
| High-density lipoprotein cholesterol | European | PRS-CS | 1 | NA | 961,631 | 0.97 (0.95-0.98) |
|  | East Asian | lassosum | 0.9 | 0.002636651 | 489,096 | 0.98 (0.97-1.00) |
| Type 2 diabetes | European | PRS-CS | 1.00E-02 | NA | 964,565 | 1.05 (1.03-1.06) |
|  | East Asian | PRS-CS | 1.00E-02 | NA | 961,902 | 1.05 (1.04-1.07) |
| Glucose level | Trans-ancestry | C+T | 0.8 | 0.5 | 534,003 | 1.02 (1.01-1.04) |

C+T, clumping and thresholding; HR, hazard ratio; CI, confidence interval.

Hazard ratios and 95% confidence intervals were estimated with adjustment for top 10 principal components and genotyping arrays.

^*^Parameter 1: linkage disequilibrium r^2^ for clumping and thresholding, s for lassosum, or phi for PRS-CS.

^†^Parameter 2: *p* value for clumping and thresholding, or lambda for lassosum.

**Table S5. Baseline characteristics of the training set**

|  | All  (n=24,251) | Women  (n=12,001) | Men  (n=12,250) |
| --- | --- | --- | --- |
| Age, years±SD | 58.2±10.7 | 57.2±10.7 | 59.2±10.6 |
| Middle school and above, % | 34.3 | 27.2 | 41.2 |
| Married, % | 85.2 | 82.2 | 88.1 |
| Family history of CVD^*^, % | 20.6 | 20.9 | 20.3 |
| Prevalent hypertension, % | 52.9 | 52.3 | 53.4 |
| Prevalent diabetes, % | 8.2 | 9.2 | 7.3 |
| Having an unfavorable lifestyle^†^, % |  |  |  |
| Current smoking | 39.0 | 6.0 | 71.4 |
| Less healthy dietary habits | 95.5 | 94.4 | 96.5 |
| Low physical activity | 49.6 | 49.4 | 49.7 |
| Unhealthy sleep duration | 33.2 | 35.1 | 31.4 |
| Unhealthy body mass index | 47.8 | 51.7 | 44.0 |
| Unhealthy waist circumference | 23.6 | 28.0 | 19.2 |
| Number of unfavorable lifestyle factors, % |  |  |  |
| 0-1 (favorable lifestyle) | 11.6 | 17.5 | 5.9 |
| 2-3 (intermediate lifestyle) | 79.3 | 76.7 | 81.9 |
| 4-5 (unfavorable lifestyle) | 9.1 | 5.8 | 12.3 |

SD, standard deviation; CVD, cardiovascular disease.

^*^Family history of CVD was defined as at least one of parents or siblings having heart disease or stroke.

^†^Unhealthy lifestyles were defined as follows: current smoking or having stopped smoking because of illness; having any of the three dietary habits (not eating fresh fruits daily, not eating vegetables daily, or eating red meat daily or less than weekly); engaging in a sex- and age-specific lower half of total physical activity; sleeping <7 or > 9 hours/day; having BMI <18.5 or ≥24.0 kg/m^2^; and having waist circumference≥90cm (men) / 85cm (women).

**Table S6. Associations of previous PRSs and disease-specific MetaPRSs with cardiovascular disease in the testing set**

|  | HR (95% CI) |
| --- | --- |
| Previous CAD PRS |  |
| PGS003725 | 1.12 (1.10-1.13) |
| PGS000337 | 1.08 (1.07-1.10) |
| PGS002262 | 1.05 (1.04-1.07) |
| PGS000013 | 1.05 (1.04-1.07) |
| Previous stroke PRS |  |
| PGS002259 | 1.07 (1.05-1.08) |
| PGS000039 | 1.05 (1.04-1.07) |
| Disease-specific MetaPRS |  |
| CVD | 1.20 (1.19-1.22) |
| CAD | 1.13 (1.11-1.14) |
| IS | 1.15 (1.14-1.17) |
| ICH | 1.13 (1.11-1.15) |

PRS, polygenic risk score; CVD, cardiovascular disease; CAD, coronary artery disease; IS, ischemic stroke; ICH, intracerebral hemorrhage; HR, hazard ratio; CI, confidence interval.

Hazard ratios and 95% confidence intervals were estimated with adjustment for sex, highest education, marital status, top 10 principal components, and genotyping arrays.

**Table S7. Associations of genetic risk and lifestyles with transition 3 after inverse probability weighting in the testing set**

|  | Genetic risk, HR (95% CI) | | |  | Lifestyles, HR (95% CI) | | |
| --- | --- | --- | --- | --- | --- | --- | --- |
|  | Low | Intermediate | High |  | Favorable | Intermediate | Unfavorable |
| Women | Reference | 1.02 (0.88-1.19) | 1.21 (1.02-1.44) |  | Reference | 1.34 (1.13-1.58) | 1.47 (1.17-1.86) |
| Men | Reference | 1.01 (0.89-1.15) | 1.16 (1.00-1.35) |  | Reference | 1.41 (1.10-1.80) | 1.58 (1.21-2.08) |

HR, hazard ratio; CI, confidence interval.

Hazard ratios and 95% confidence intervals were estimated with adjustment for highest education, marital status, and top 10 principal components and genotyping arrays in the analysis of genetic risk or study regions in the analysis of lifestyles.

The genetic risk and lifestyles were categorized in the same way as in Table 2.

**Table S8. Associations of joint categories of genetic risk and lifestyles with different transitions in women in the testing set**

| Genetic risk | Lifestyles | Baseline → CVD | | |  | Baseline → non-CVD death | | |  | CVD → death | | |  |
| --- | --- | --- | --- | --- | --- | --- | --- | --- | --- | --- | --- | --- | --- |
|  |  | HR (95% CI) | *p* | Adjusted *p^*^* |  | HR (95% CI) | *p* | Adjusted *p^*^* |  | HR (95% CI) | *p* | Adjusted *p^*^* |  |
| Low | Favorable | Reference |  |  |  | Reference |  |  |  | Reference |  |  |  |
|  | Intermediate | 1.08 (0.97-1.22) | 0.174 | 1 |  | 0.84 (0.63-1.14) | 0.263 | 1 |  | 1.27 (0.91-1.77) | 0.14 | 1 |  |
|  | Unfavorable | 1.31 (1.06-1.61) | 0.011 | 0.202 |  | 0.79 (0.41-1.50) | 0.466 | 1 |  | 1.06 (0.62-1.80) | 0.798 | 1 |  |
| Intermediate | Favorable | 1.10 (0.98-1.24) | 0.113 | 1 |  | 0.82 (0.60-1.12) | 0.209 | 1 |  | 1.27 (0.89-1.81) | 0.154 | 1 |  |
|  | Intermediate | 1.32 (1.19-1.48) | 4.2E-07 | 7.6E-06 |  | 0.89 (0.68-1.17) | 0.411 | 1 |  | 1.29 (0.93-1.78) | 0.099 | 1 |  |
|  | Unfavorable | 1.68 (1.46-1.94) | 9.9E-13 | 1.8E-11 |  | 0.83 (0.53-1.29) | 0.401 | 1 |  | 1.49 (1.02-2.19) | 0.032 | 0.574 |  |
| High | Favorable | 1.36 (1.18-1.58) | 3.4E-05 | 6E-04 |  | 0.97 (0.65-1.44) | 0.88 | 1 |  | 0.90 (0.57-1.41) | 0.676 | 1 |  |
|  | Intermediate | 1.81 (1.62-2.03) | 5.6E-25 | 1E-23 |  | 0.95 (0.71-1.29) | 0.754 | 1 |  | 1.59 (1.14-2.21) | 0.005 | 0.085 |  |
|  | Unfavorable | 2.30 (1.93-2.74) | 1.1E-20 | 2E-19 |  | 1.18 (0.67-2.07) | 0.559 | 1 |  | 1.93 (1.26-2.97) | 0.002 | 0.038 |  |

CVD, cardiovascular disease; HR, hazard ratio; CI, confidence interval.

^*^Bonferroni-corrected *p*-values were calculated by multiplying the original *p*-values by 18.

Hazard ratios and 95% confidence intervals were estimated with adjustment for highest education, marital status, top 10 principal components, and genotyping arrays.

The genetic risk and lifestyles were categorized in the same way as in Table 2.

**Table S9. Associations of joint categories of genetic risk and lifestyles with different transitions in men in the testing set**

| Genetic risk | Lifestyles | Baseline → CVD | | |  | Baseline → non-CVD death | | |  | CVD → death | | |  |
| --- | --- | --- | --- | --- | --- | --- | --- | --- | --- | --- | --- | --- | --- |
|  |  | HR (95% CI) | *p* | Adjusted *p^*^* |  | HR (95% CI) | *p* | Adjusted *p^*^* |  | HR (95% CI) | *p* | Adjusted *p^*^* |  |
| Low | Favorable | Reference |  |  |  | Reference |  |  |  | Reference |  |  |  |
|  | Intermediate | 1.22 (0.97-1.53) | 0.082 | 1 |  | 1.07 (0.71-1.62) | 0.743 | 1 |  | 1.76 (1.03-3.00) | 0.032 | 0.574 |  |
|  | Unfavorable | 1.71 (1.32-2.20) | 4E-05 | 7.1E-04 |  | 1.03 (0.61-1.73) | 0.925 | 1 |  | 2.00 (1.12-3.56) | 0.017 | 0.3 |  |
| Intermediate | Favorable | 1.19 (0.93-1.53) | 0.164 | 1 |  | 0.79 (0.49-1.28) | 0.331 | 1 |  | 1.25 (0.70-2.24) | 0.45 | 1 |  |
|  | Intermediate | 1.50 (1.20-1.87) | 3.1E-04 | 0.006 |  | 1.11 (0.74-1.67) | 0.602 | 1 |  | 1.76 (1.04-2.99) | 0.029 | 0.518 |  |
|  | Unfavorable | 2.18 (1.74-2.75) | 2.5E-11 | 4.6E-10 |  | 1.09 (0.70-1.70) | 0.712 | 1 |  | 2.01 (1.17-3.46) | 0.01 | 0.174 |  |
| High | Favorable | 1.52 (1.12-2.05) | 0.006 | 0.116 |  | 0.60 (0.30-1.24) | 0.168 | 1 |  | 1.43 (0.74-2.79) | 0.283 | 1 |  |
|  | Intermediate | 2.01 (1.60-2.51) | 1.1E-09 | 1.9E-08 |  | 1.05 (0.69-1.60) | 0.818 | 1 |  | 1.99 (1.17-3.39) | 0.01 | 0.171 |  |
|  | Unfavorable | 2.75 (2.16-3.51) | 3.8E-16 | 6.8E-15 |  | 1.06 (0.62-1.80) | 0.83 | 1 |  | 2.30 (1.30-4.05) | 0.003 | 0.06 |  |

CVD, cardiovascular disease; HR, hazard ratio; CI, confidence interval.

^*^Bonferroni-corrected *p*-values were calculated by multiplying the original *p*-values by 18.

Hazard ratios and 95% confidence intervals were estimated with adjustment for highest education, marital status, top 10 principal components, and genotyping arrays.

The genetic risk and lifestyles were categorized in the same way as in Table 2.

**Table S10. Differences in life expectancy at age 40 with and without cardiovascular disease between each joint category of genetic risk and lifestyles and the highest risk group in the testing set**

| Genetic risk | Lifestyles | Women | |  | Men | |
| --- | --- | --- | --- | --- | --- | --- |
|  |  | Total, y (95% CI) | Free of CVD, y (95% CI) |  | Total, y (95% CI) | Free of CVD, y (95% CI) |
| High | Unfavorable | Reference | Reference |  | Reference | Reference |
|  | Intermediate | 2.3 (-1.1-5.6) | 2.5 (0.4-4.6) |  | 2.2 (-2.6-6.7) | 2.9 (-0.2-6.0) |
|  | Favorable | 5.9 (2.3-9.3) | 5.3 (3.0-7.6) |  | 6.1 (0.8-10.7) | 6.2 (2.4-9.8) |
| Intermediate | Unfavorable | 3.0 (-0.6-6.5) | 3.4 (1.1-5.7) |  | 1.7 (-3.2-6.3) | 2.1 (-1.1-5.2) |
|  | Intermediate | 4.5 (1.2-7.8) | 5.7 (3.6-7.8) |  | 3.9 (-1.0-8.2) | 5.4 (2.3-8.4) |
|  | Favorable | 5.3 (1.9-8.5) | 7.6 (5.5-9.7) |  | 7.1 (2.2-11.1) | 8.0 (4.7-11.2) |
| Low | Unfavorable | 5.7 (1.7-9.3) | 6.0 (3.2-8.7) |  | 2.9 (-1.9-7.4) | 4.4 (0.9-7.7) |
|  | Intermediate | 5.3 (2.0-8.5) | 7.7 (5.6-9.8) |  | 4.8 (0.0-9.0) | 7.2 (4.0-10.3) |
|  | Favorable | 6.2 (3.1-9.0) | 8.3 (6.5-10.0) |  | 7.8 (3.8-11.1) | 9.1 (6.6-11.4) |

CI, confidence interval; CVD, cardiovascular disease.

The genetic risk and lifestyles were categorized in the same way as in Table 2.


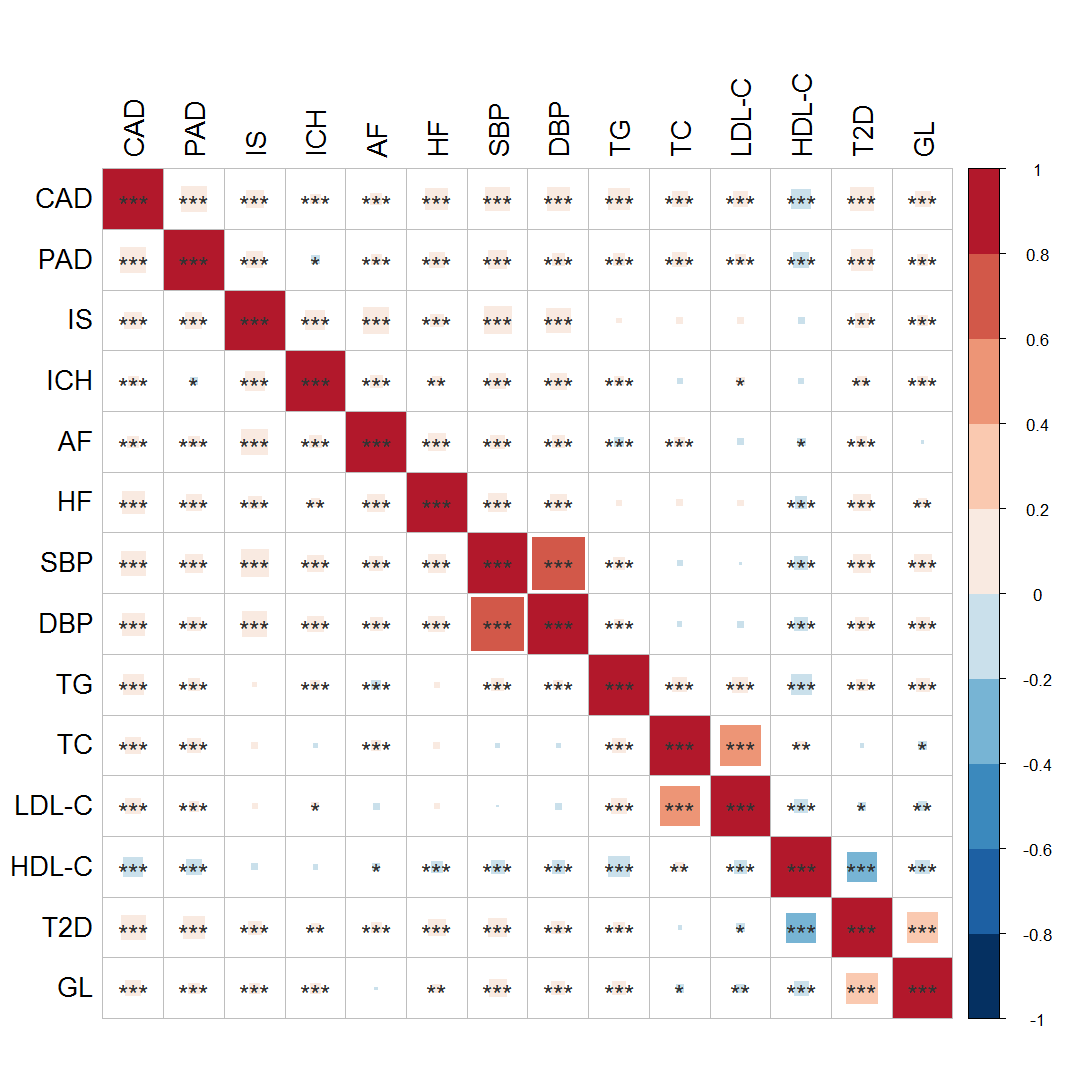


**Fig S1. Correlations among trait-specific PRSs in the training set**

AF, atrial fibrillation; CAD, coronary artery disease; DBP, diastolic blood pressure; GL, glucose level; HDL-C, high-density lipoprotein cholesterol; HF, heart failure; ICH, intracerebral hemorrhage; IS, ischemic stroke; LDL-C, low-density lipoprotein cholesterol; PAD, peripheral arterial disease; SBP, systolic blood pressure; TC, total cholesterol; TG, triglycerides; T2D, type 2 diabetes.

* *p*<0.05, ** *p*<0.01, *** *p*<0.001. The color and size of the squares indicate the magnitude of correlation coefficients between the PRS pairs. Correlation coefficients and *p* values were estimated from Pearson correlation tests.

**
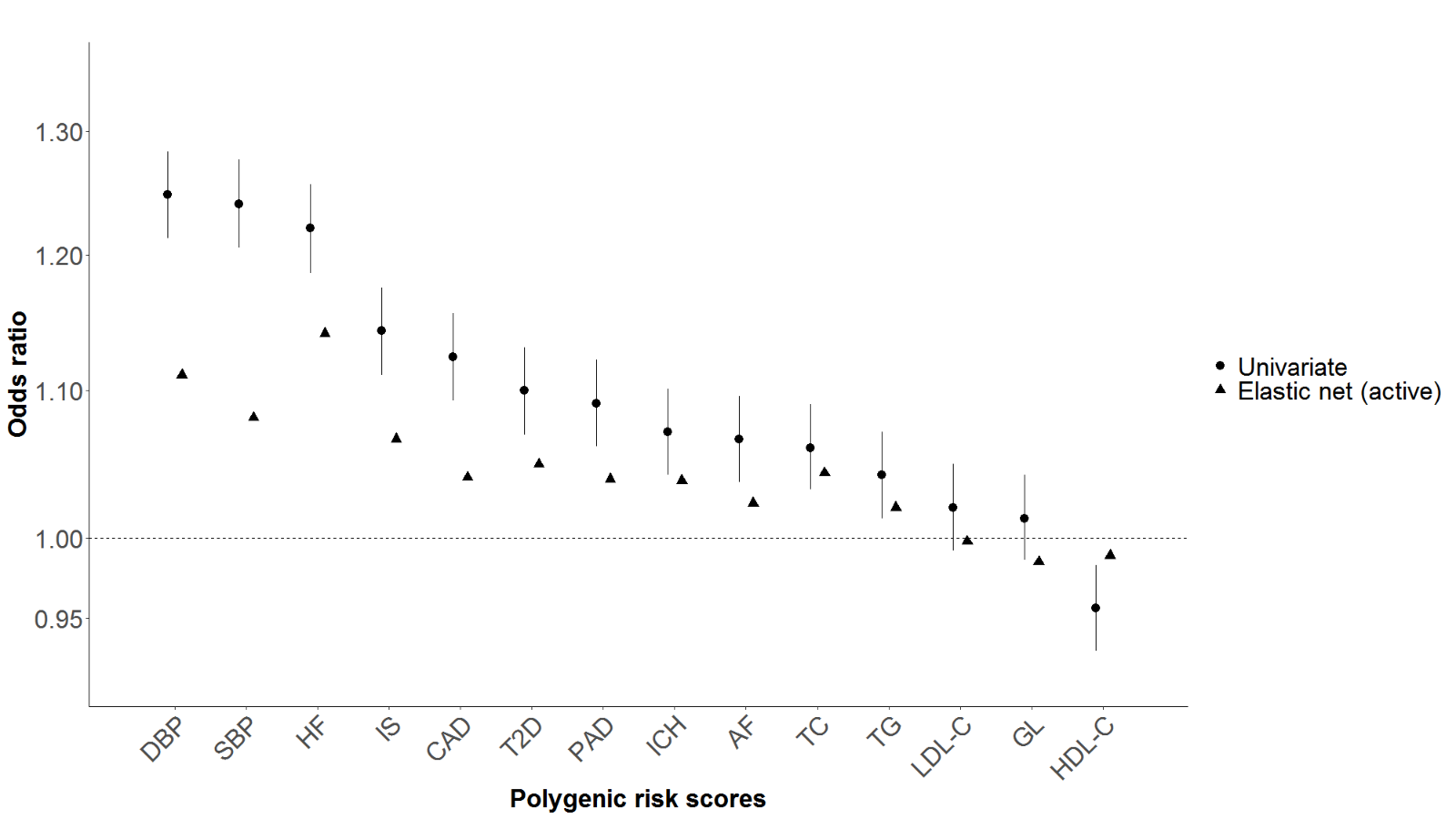
**

**Fig S2. Associations of trait-specific PRSs (per standard deviation increment) with cardiovascular disease in the training set**

AF, atrial fibrillation; CAD, coronary artery disease; DBP, diastolic blood pressure; GL, glucose level; HDL-C, high-density lipoprotein cholesterol; HF, heart failure; ICH, intracerebral hemorrhage; IS, ischemic stroke; LDL-C, low-density lipoprotein cholesterol; PAD, peripheral arterial disease; SBP, systolic blood pressure; TC, total cholesterol; TG, triglycerides; T2D, type 2 diabetes.

The odds ratio of each trait-specific PRS were estimated from either univariate logistic models (shown as filled circles) or elastic-net logistic models (shown as filled triangles). The 95% confidence intervals were only available for univariate logistic models. For elastic-net, 'active' indicates that the odds ratios are < 0.999 or > 1.001, shown as filled triangles.

**
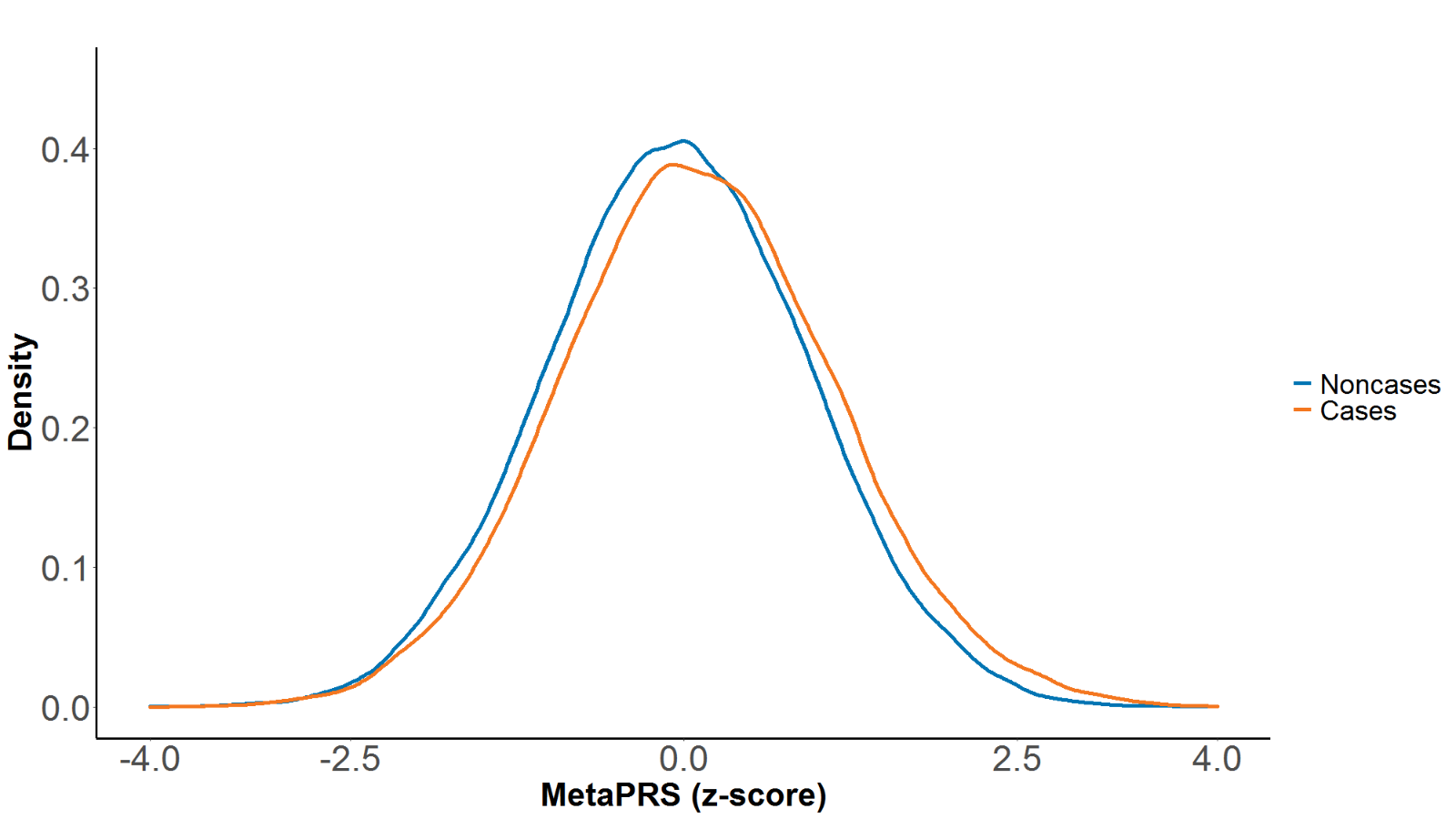
**

**Fig S3. Density plot of the MetaPRS in the testing set**

**
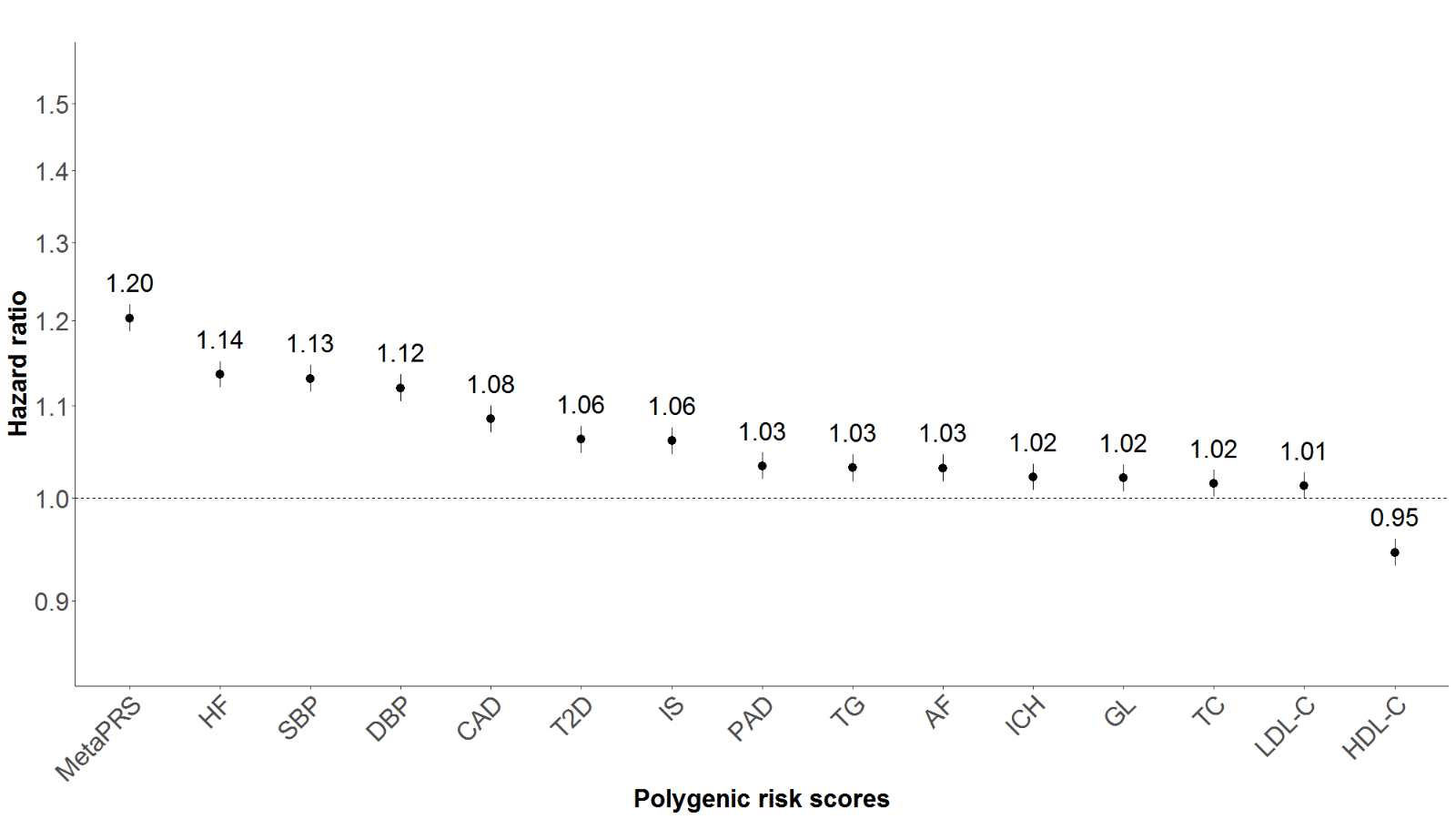
**

**Fig S4. Associations of the MetaPRS and trait-specific PRSs (per standard deviation increment) with cardiovascular disease in the testing set**

AF, atrial fibrillation; CAD, coronary artery disease; DBP, diastolic blood pressure; GL, glucose level; HDL-C, high-density lipoprotein cholesterol; HF, heart failure; ICH, intracerebral hemorrhage; IS, ischemic stroke; LDL-C, low-density lipoprotein cholesterol; PAD, peripheral arterial disease; SBP, systolic blood pressure; TC, total cholesterol; TG, triglycerides; T2D, type 2 diabetes.

Hazard ratios and 95% confidence intervals were estimated with adjustment for sex, highest education, marital status, top 10 principal components, and genotyping arrays.


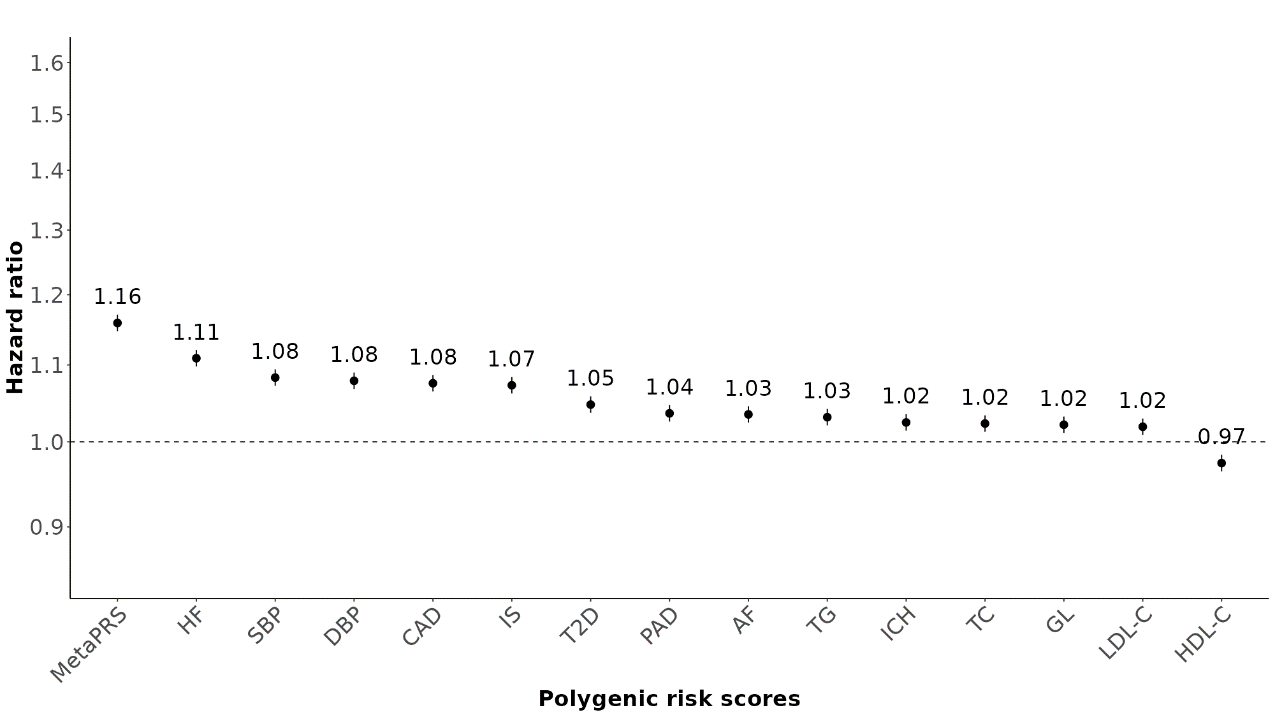


**Fig S5. Sensitivity analysis of building the MetaPRS using trans-ancestry GWAS summary statistics**

AF, atrial fibrillation; CAD, coronary artery disease; DBP, diastolic blood pressure; GL, glucose level; HDL-C, high-density lipoprotein cholesterol; HF, heart failure; ICH, intracerebral hemorrhage; IS, ischemic stroke; LDL-C, low-density lipoprotein cholesterol; PAD, peripheral arterial disease; SBP, systolic blood pressure; TC, total cholesterol; TG, triglycerides; T2D, type 2 diabetes.

Hazard ratios and 95% confidence intervals were estimated with adjustment for sex, highest education, marital status, top 10 principal components, and genotyping arrays.


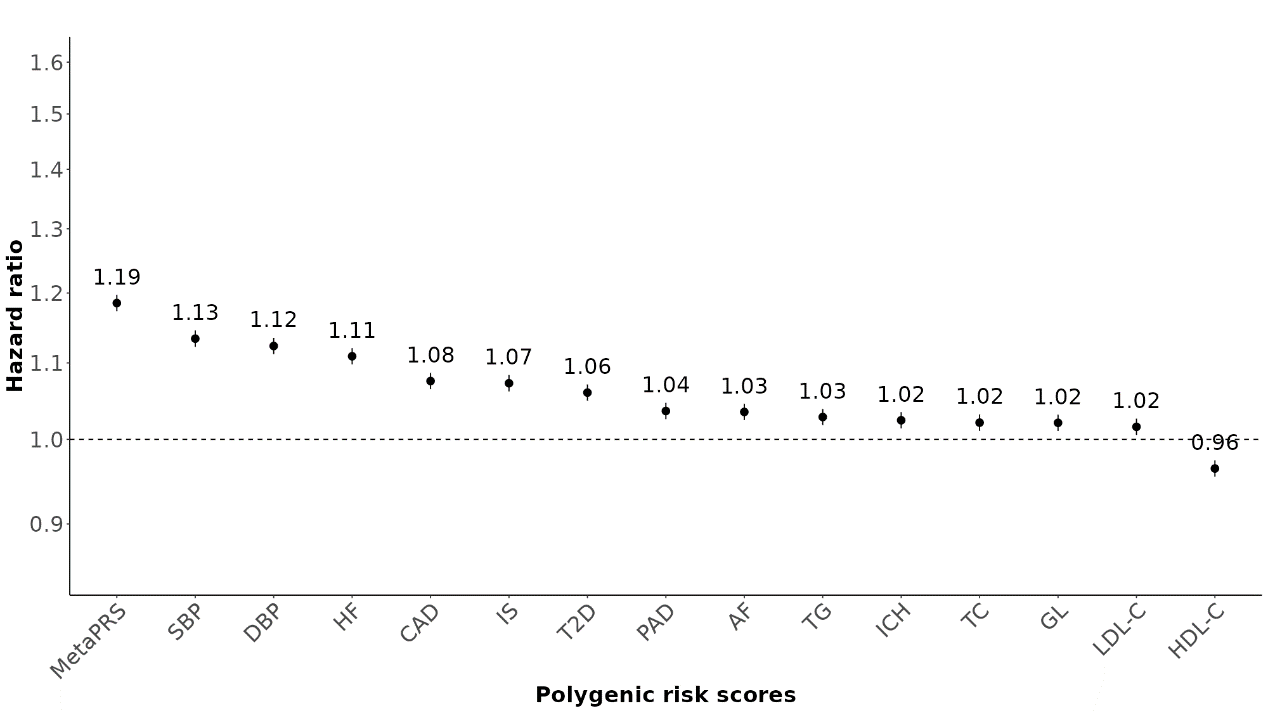


**Fig S6. Sensitivity analysis of building the MetaPRS including rare variants**

AF, atrial fibrillation; CAD, coronary artery disease; DBP, diastolic blood pressure; GL, glucose level; HDL-C, high-density lipoprotein cholesterol; HF, heart failure; ICH, intracerebral hemorrhage; IS, ischemic stroke; LDL-C, low-density lipoprotein cholesterol; PAD, peripheral arterial disease; SBP, systolic blood pressure; TC, total cholesterol; TG, triglycerides; T2D, type 2 diabetes.

Hazard ratios and 95% confidence intervals were estimated with adjustment for sex, highest education, marital status, top 10 principal components, and genotyping arrays.


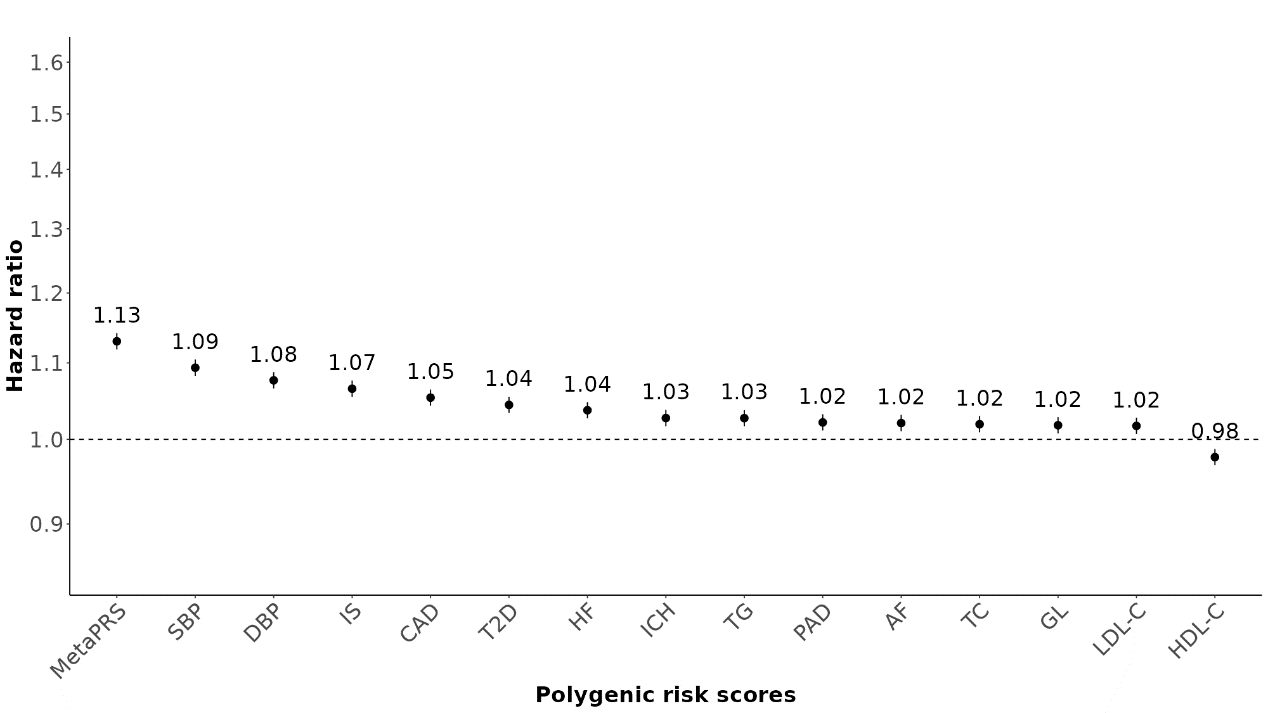


**Fig S7. Sensitivity analysis of building the MetaPRS using East Asian GWAS only**

AF, atrial fibrillation; CAD, coronary artery disease; DBP, diastolic blood pressure; GL, glucose level; HDL-C, high-density lipoprotein cholesterol; HF, heart failure; ICH, intracerebral hemorrhage; IS, ischemic stroke; LDL-C, low-density lipoprotein cholesterol; PAD, peripheral arterial disease; SBP, systolic blood pressure; TC, total cholesterol; TG, triglycerides; T2D, type 2 diabetes.

Hazard ratios and 95% confidence intervals were estimated with adjustment for sex, highest education, marital status, top 10 principal components, and genotyping arrays.

**
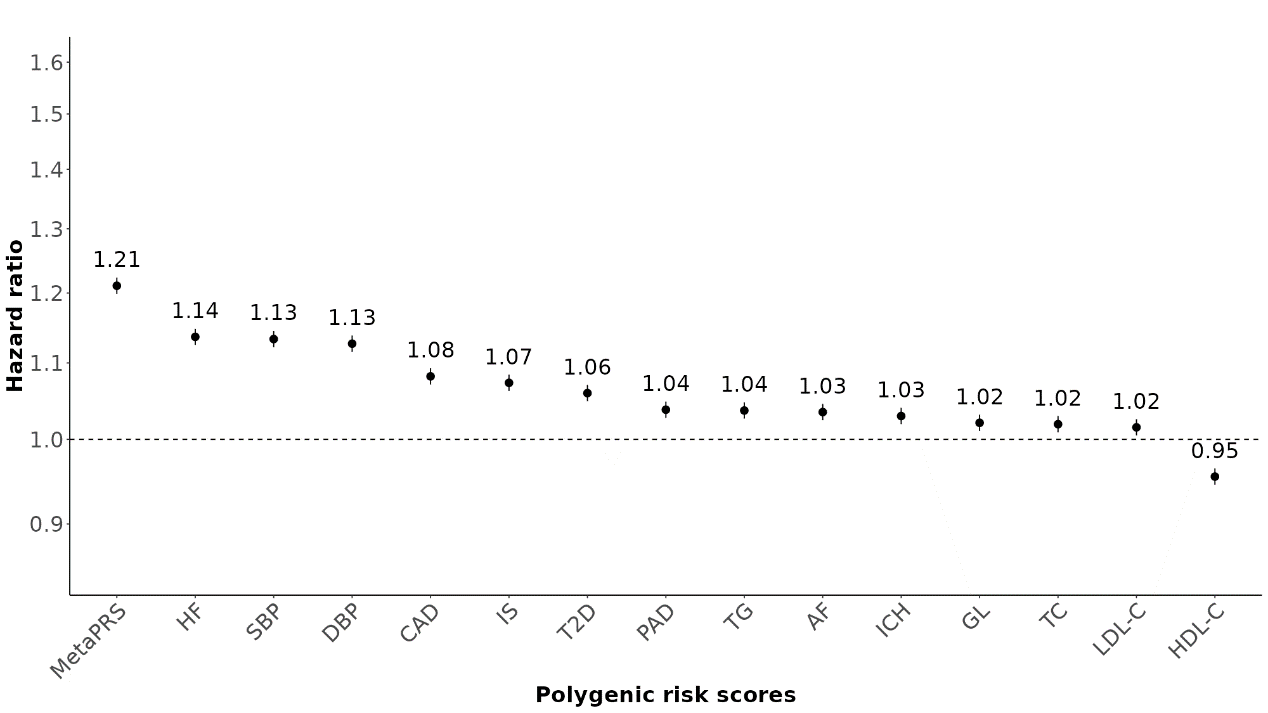
**

**Fig S8. Sensitivity analysis of building the MetaPRS in the training set without COPD cases**

AF, atrial fibrillation; CAD, coronary artery disease; DBP, diastolic blood pressure; GL, glucose level; HDL-C, high-density lipoprotein cholesterol; HF, heart failure; ICH, intracerebral hemorrhage; IS, ischemic stroke; LDL-C, low-density lipoprotein cholesterol; PAD, peripheral arterial disease; SBP, systolic blood pressure; TC, total cholesterol; TG, triglycerides; T2D, type 2 diabetes.

Hazard ratios and 95% confidence intervals were estimated with adjustment for sex, highest education, marital status, top 10 principal components, and genotyping arrays.


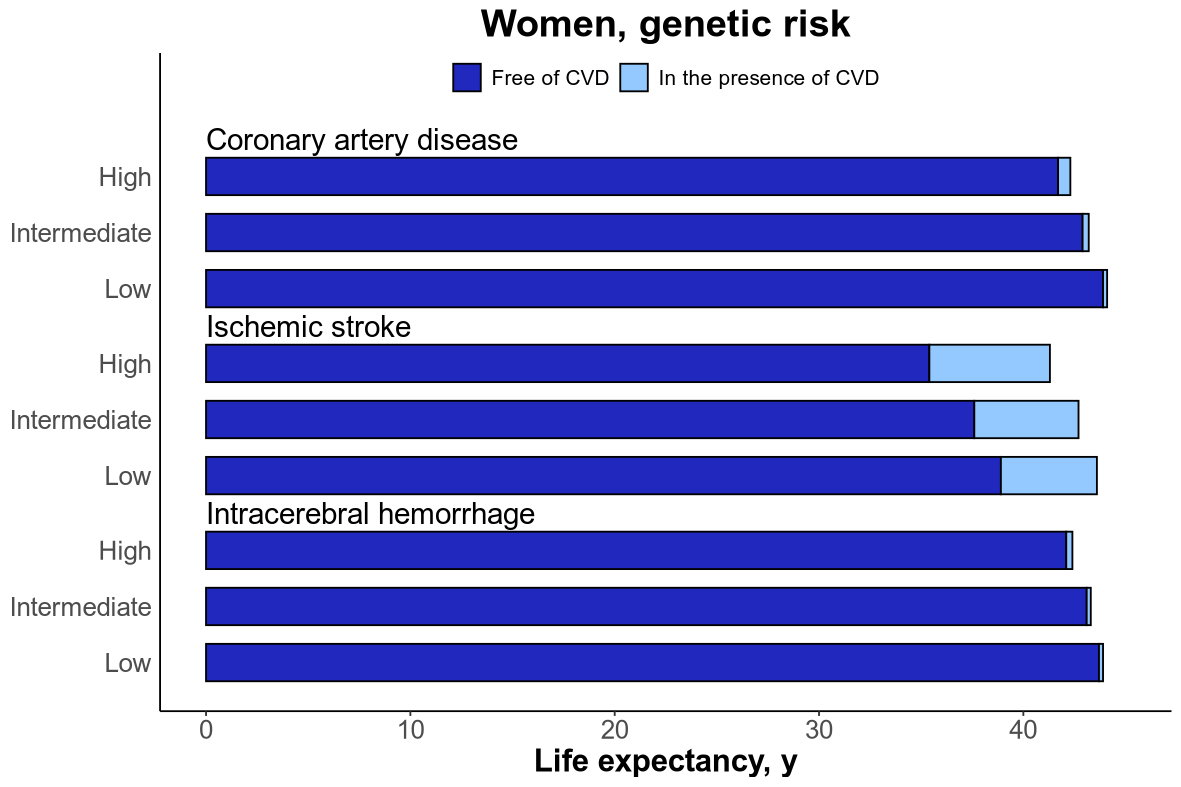

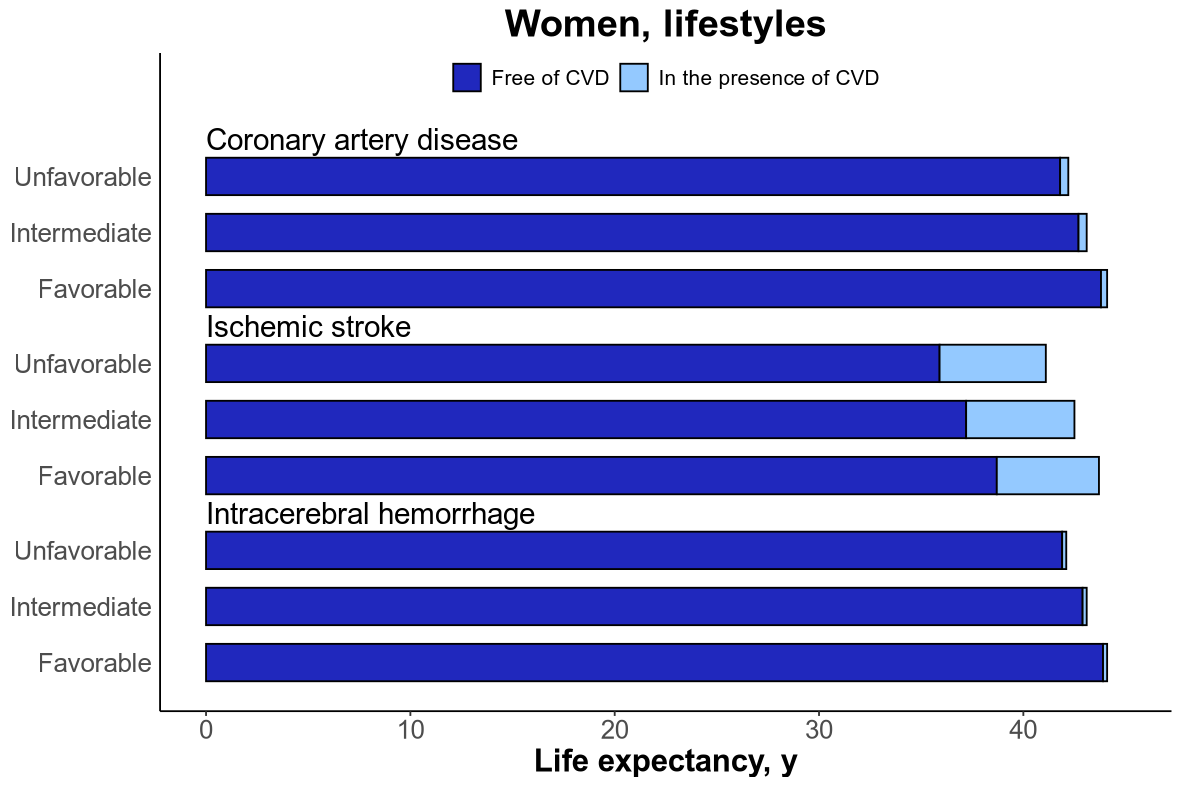
**
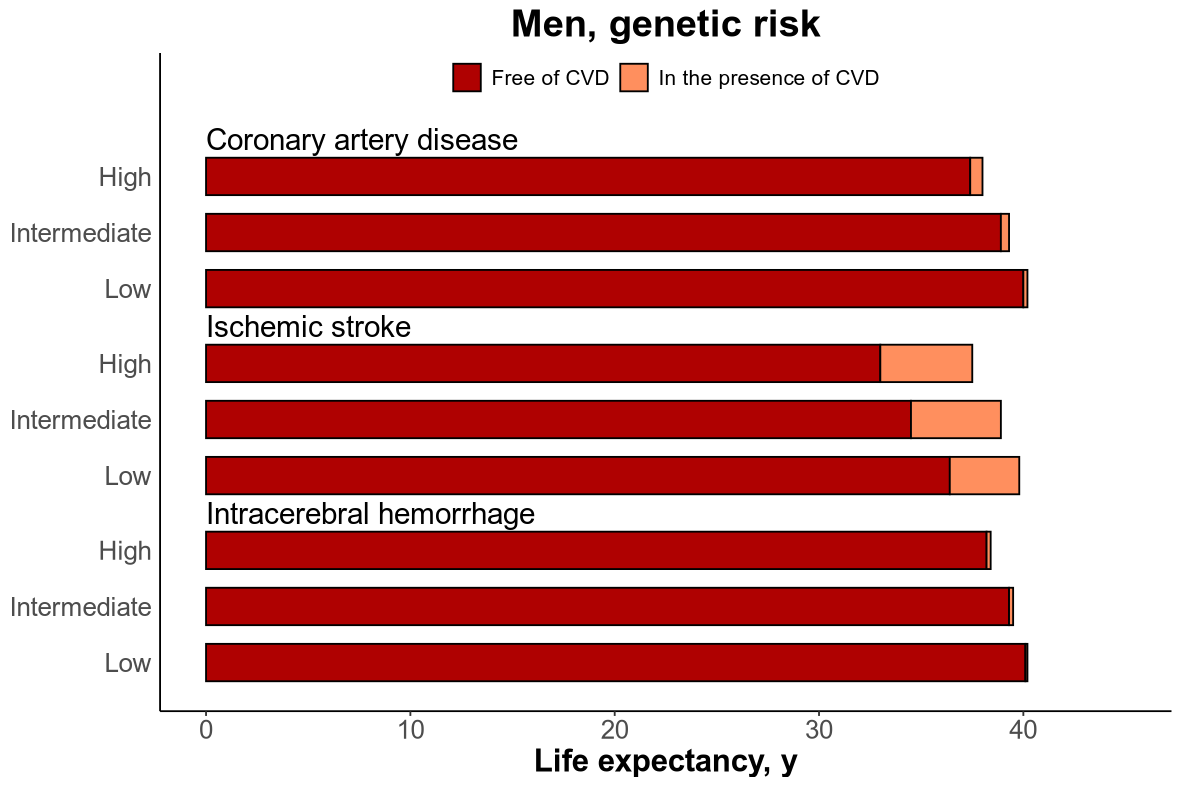
**
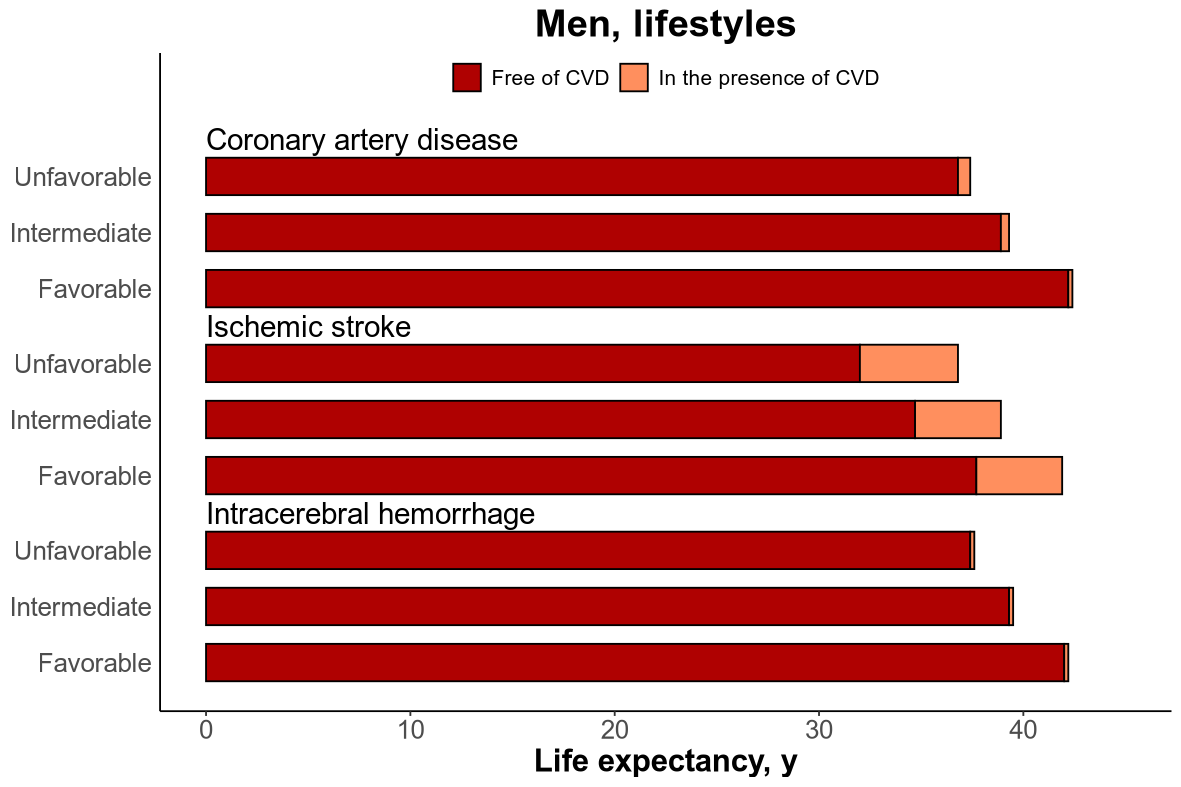


**Fig S9. Life expectancy at age 40 with and without cardiovascular disease subtypes according to genetic risk and lifestyles in the testing set**

CVD, cardiovascular disease.

The genetic risk and lifestyles were categorized in the same way as in Table 2.


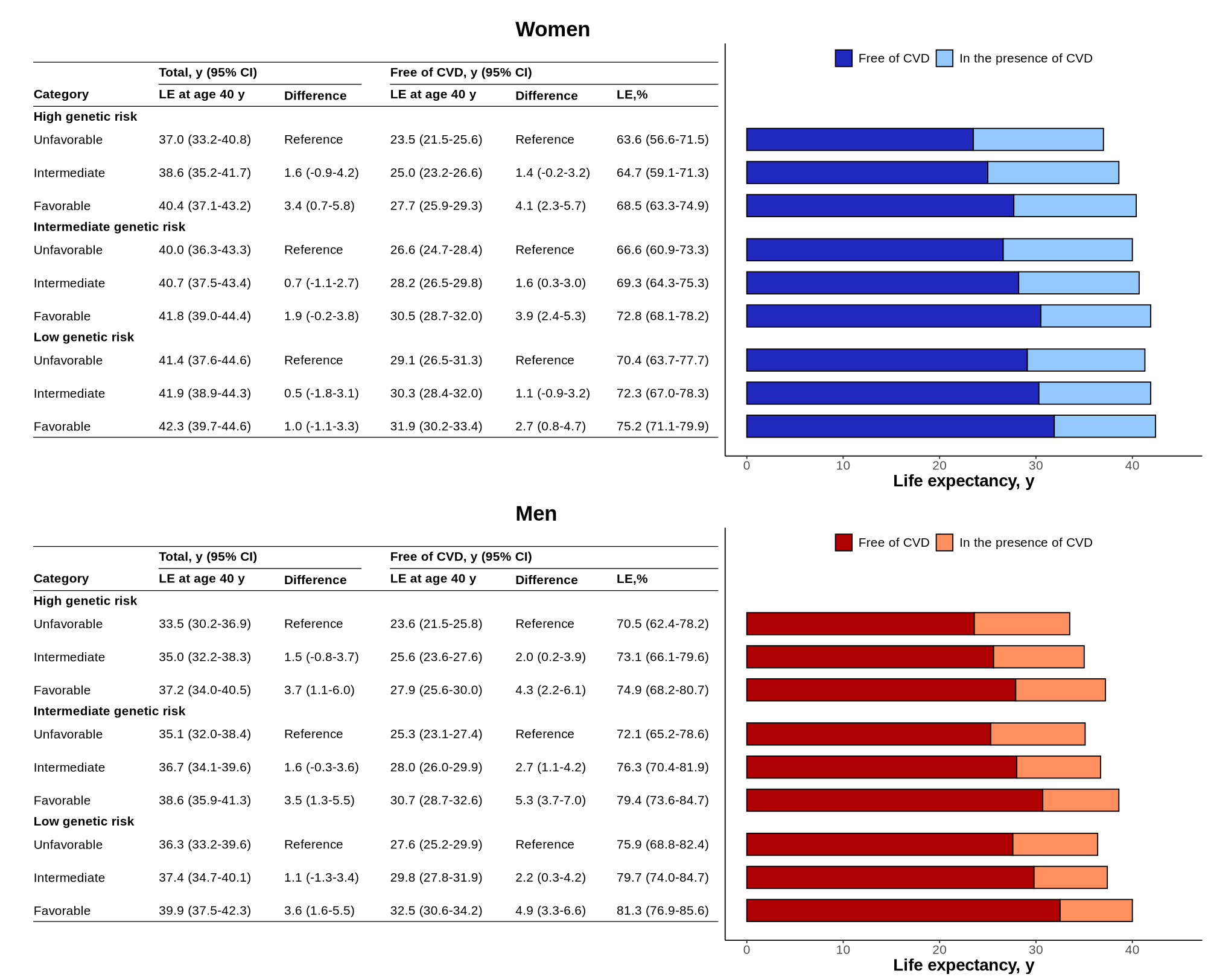


**Fig S10. Life expectancy at age 40 with and without cardiovascular disease according to joint categories of genetic risk and lifestyles (excluding diet factors) in the testing set**

CI, confidence interval; LE, life expectancy; CVD, cardiovascular disease.

The genetic risk was categorized into low (bottom quintile), intermediate (2nd-4th quintile), and high (top quintile) according to quintile cutoff points of the MetaPRS. The lifestyles were categorized into favorable (0-1), intermediate (2-3), and unfavorable (4-5) according to the number of unfavorable lifestyle factors.

**
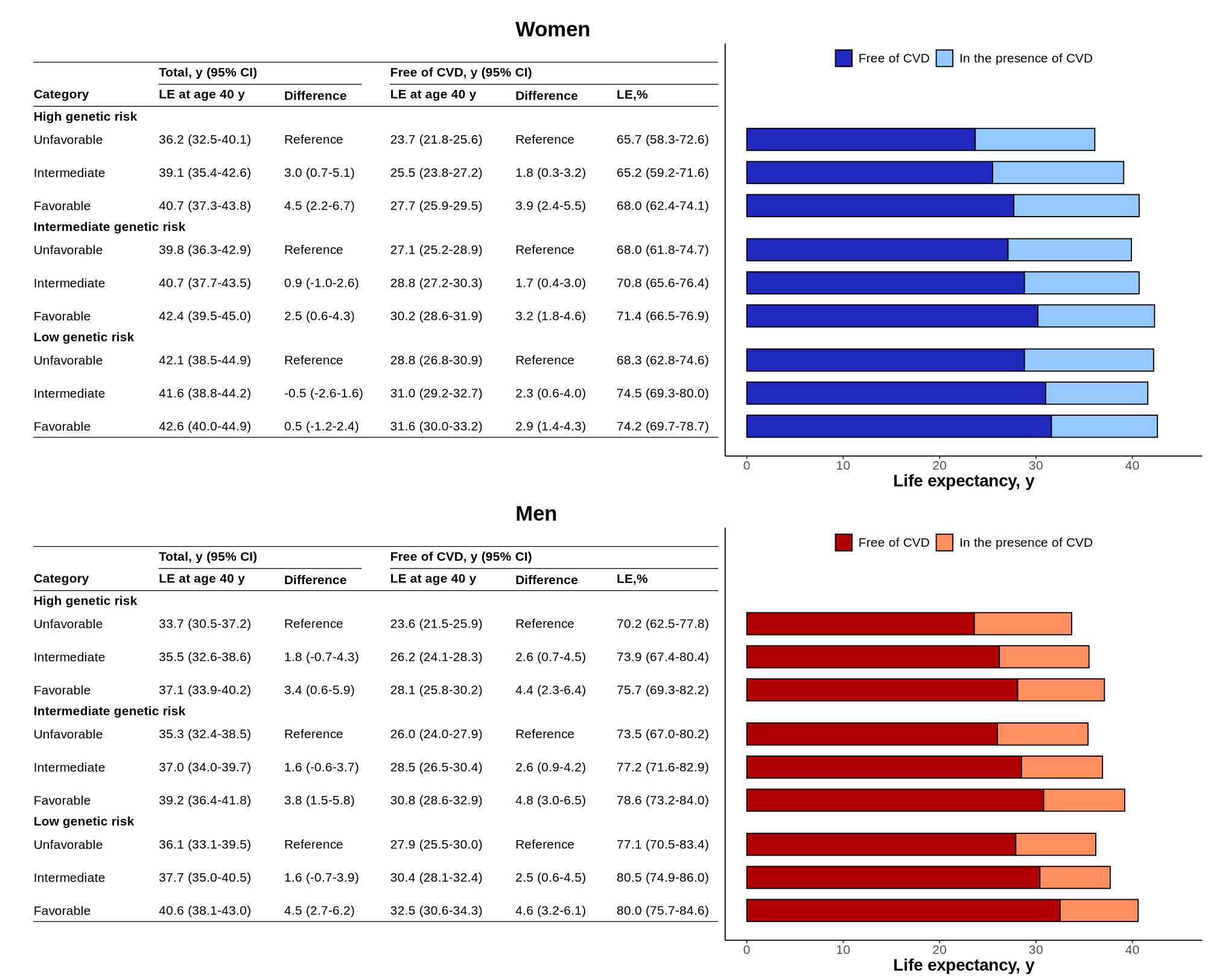
**

**Fig S11. Life expectancy at age 40 with and without cardiovascular disease according to joint categories of genetic risk and lifestyles (with specific diet habits as single lifestyle factors) in the testing set**

CI, confidence interval; LE, life expectancy; CVD, cardiovascular disease.

The genetic risk was categorized into low (bottom quintile), intermediate (2nd-4th quintile), and high (top quintile) according to quintile cutoff points of the MetaPRS. The lifestyles were categorized into favorable (0-3), intermediate (4-5), and unfavorable (6-8) according to the number of unfavorable lifestyle factors.


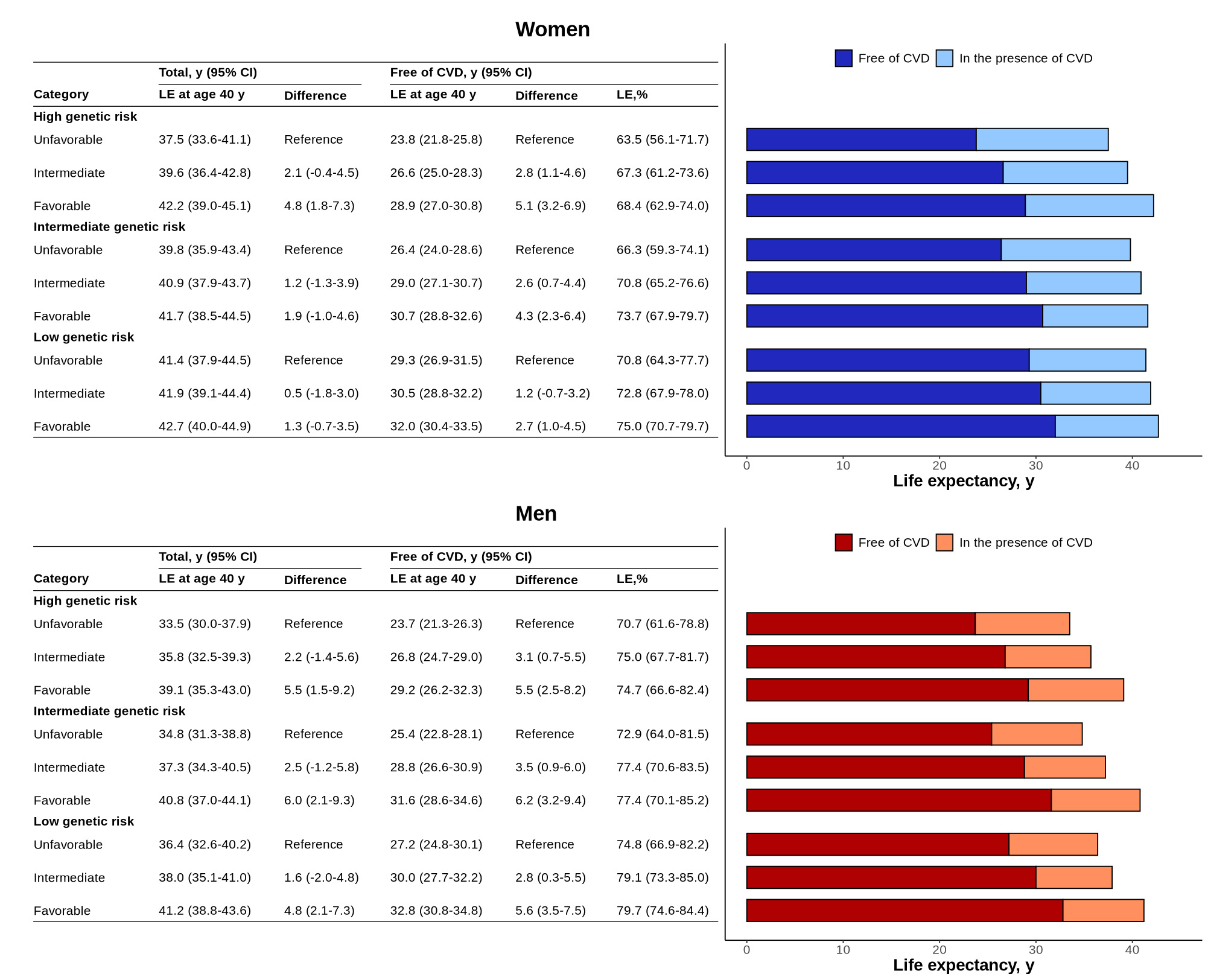


**Fig S12. Life expectancy at age 40 with and without cardiovascular disease according to joint categories of genetic risk (categorized by tertiles of the MetaPRS) and lifestyles in the testing set**

CI, confidence interval; LE, life expectancy.

The genetic risk was categorized into low (bottom tertile), intermediate (middle tertile), and high (top tertile) according to quintile cutoff points of the MetaPRS. The lifestyles were categorized into favorable (0-1), intermediate (2-4), and unfavorable (5-6) according to the number of unfavorable lifestyle factors.

**
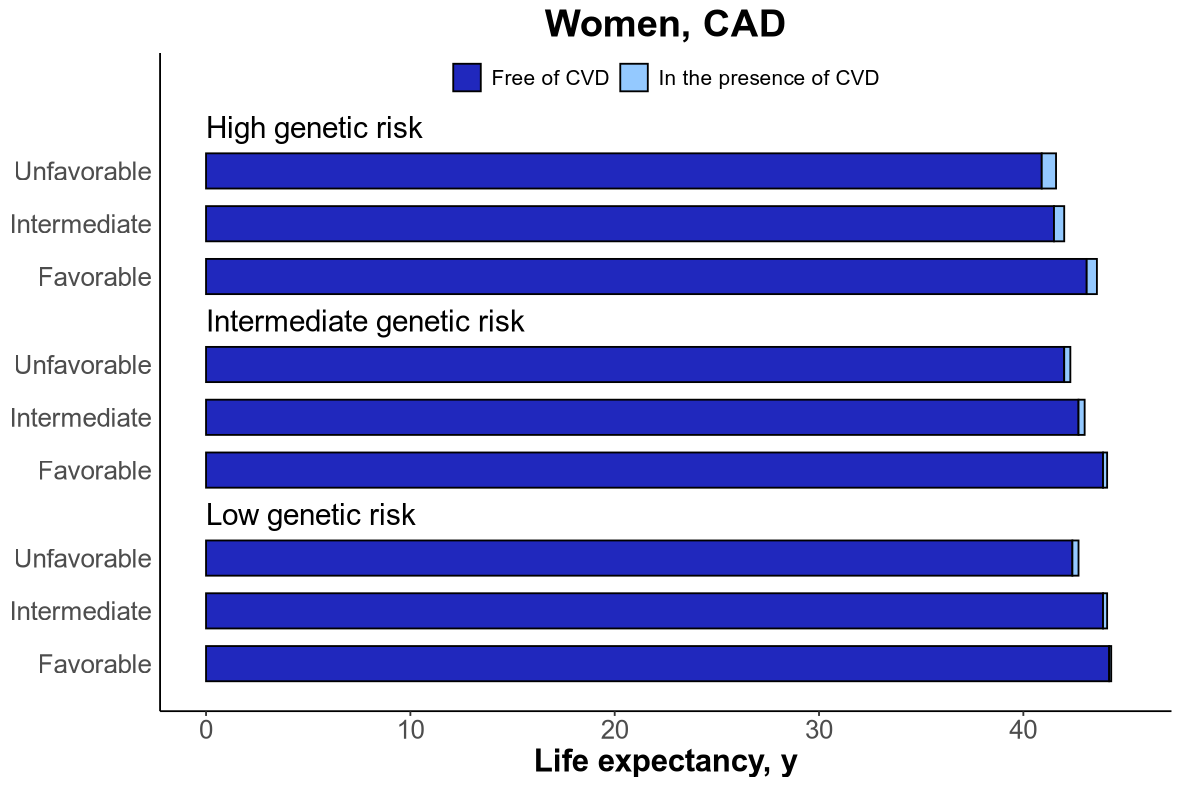

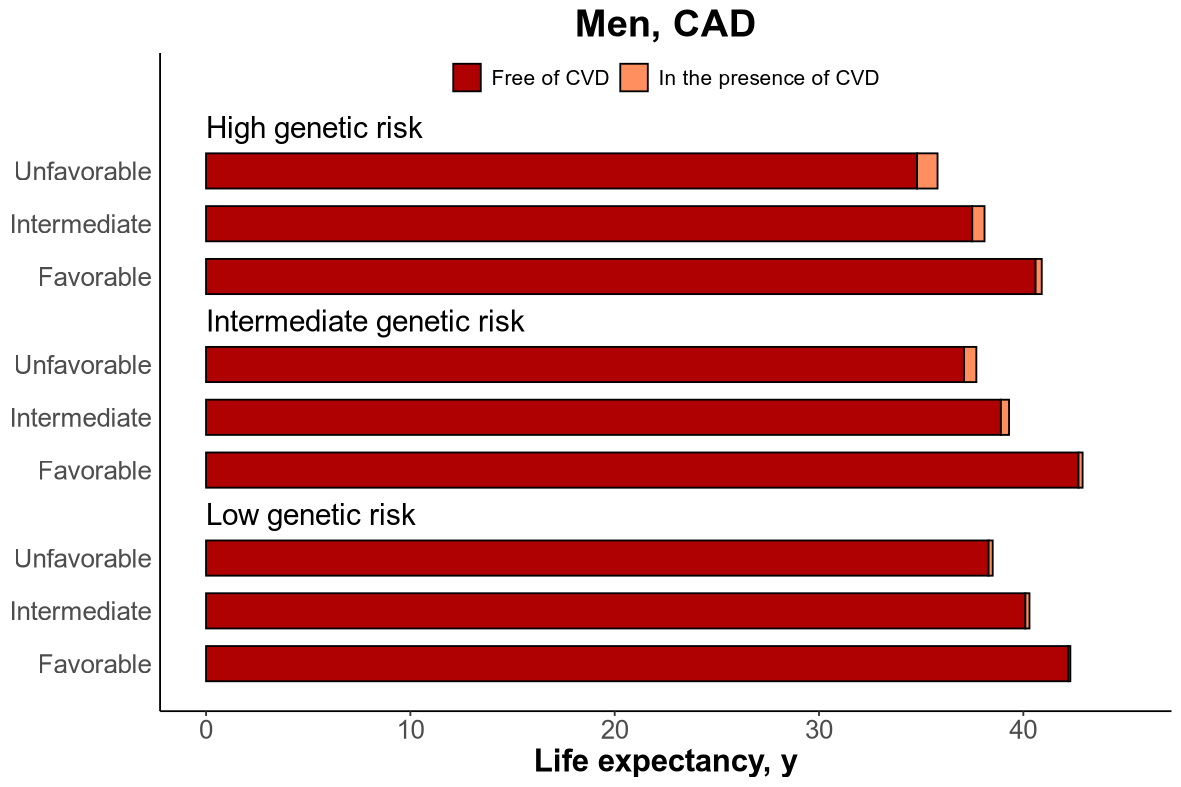

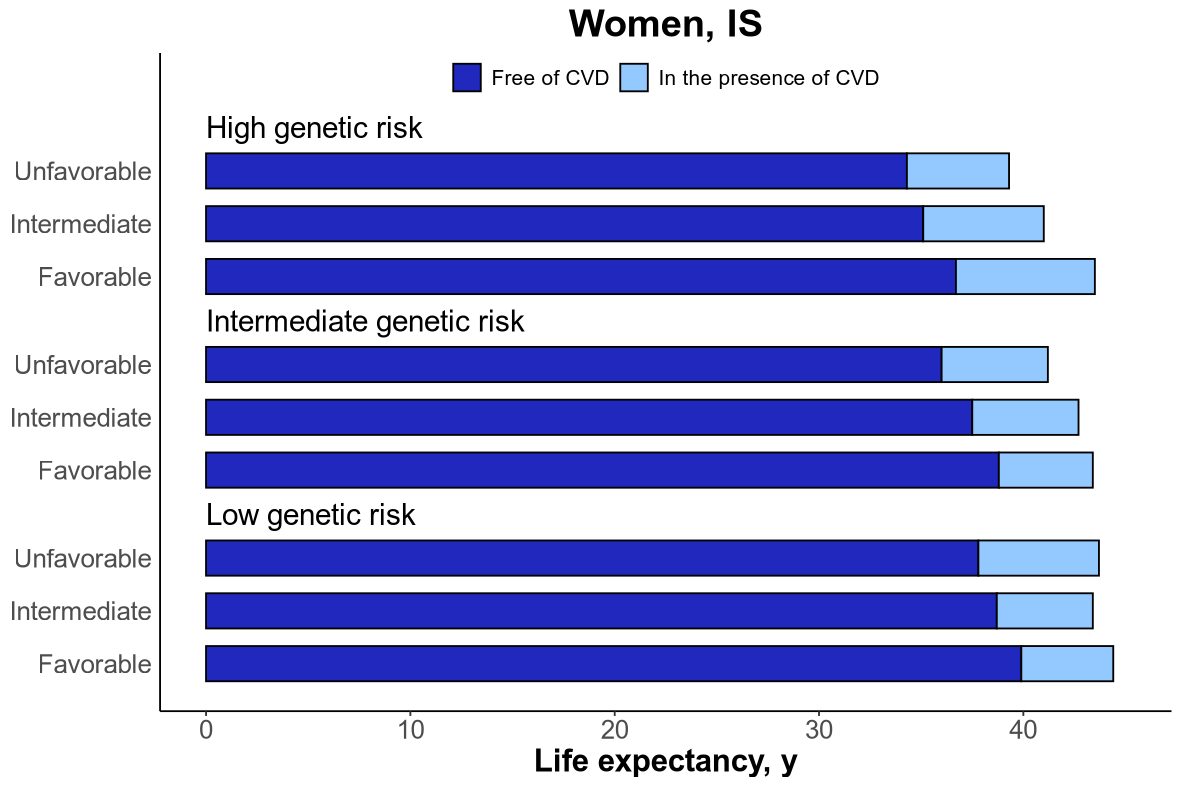

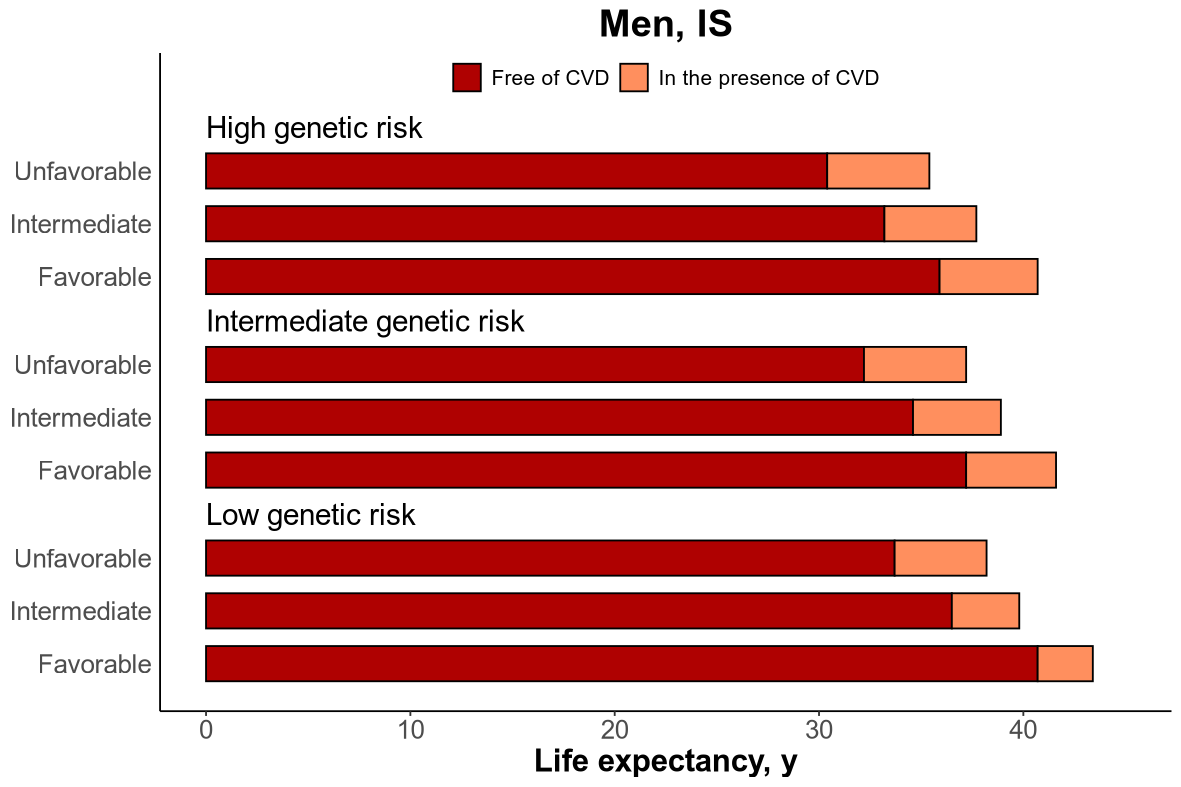

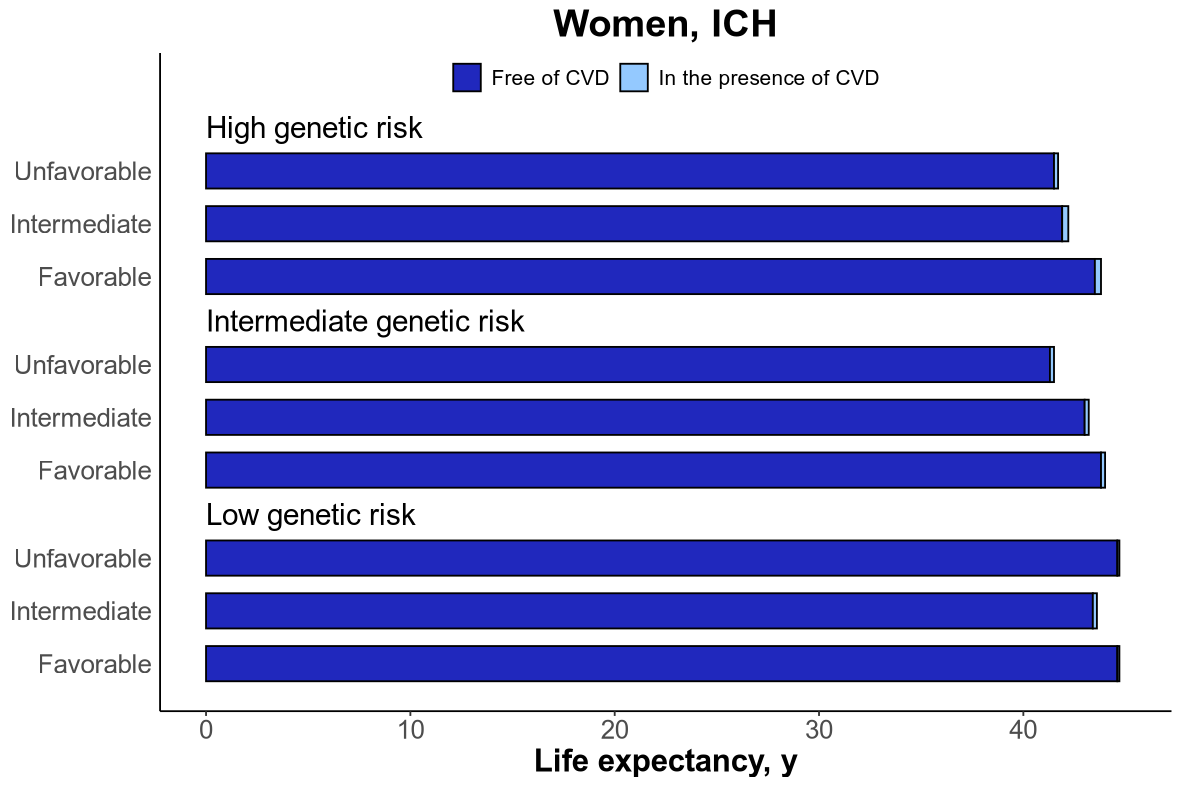

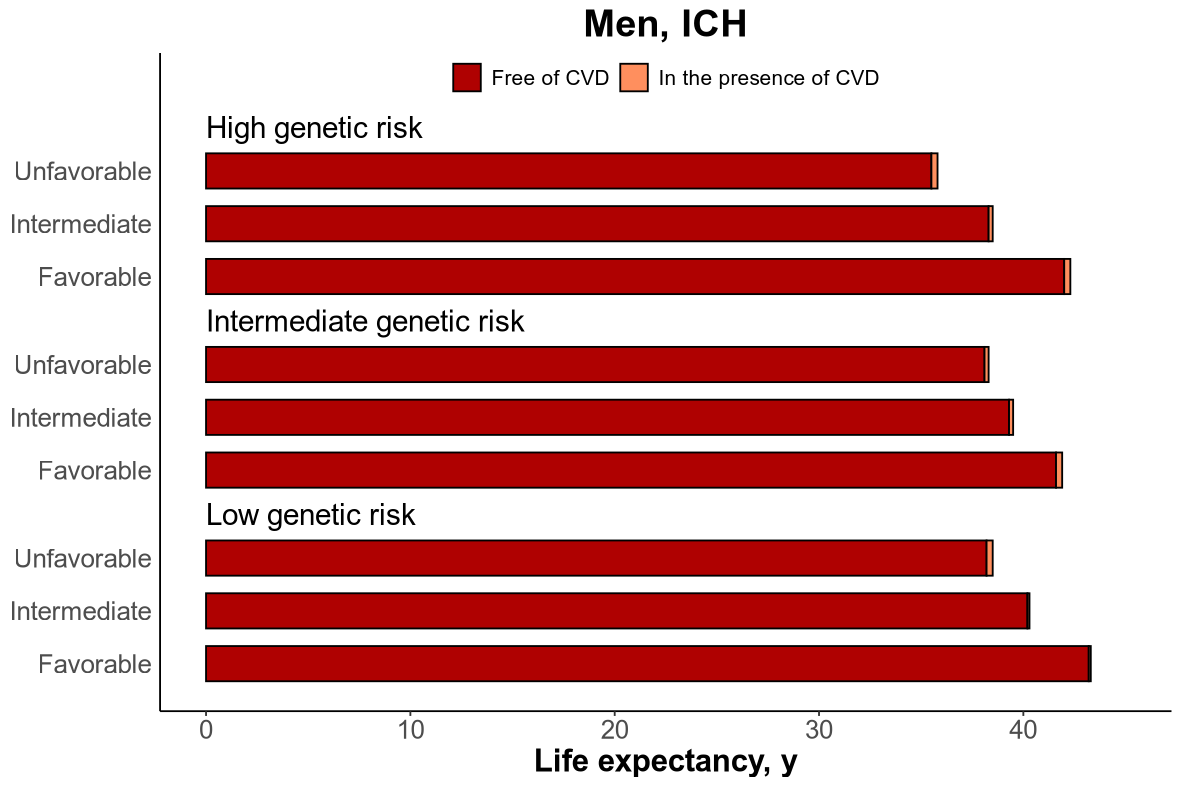
**

**Fig S13. Life expectancy at age 40 with and without cardiovascular disease subtypes according to joint categories of genetic risk and lifestyles in the testing set**

CAD, coronary artery disease; CVD, cardiovascular disease; IS, ischemic stroke; ICH, intracerebral hemorrhage.

The genetic risk and lifestyles were categorized in the same way as in Table 2.

**
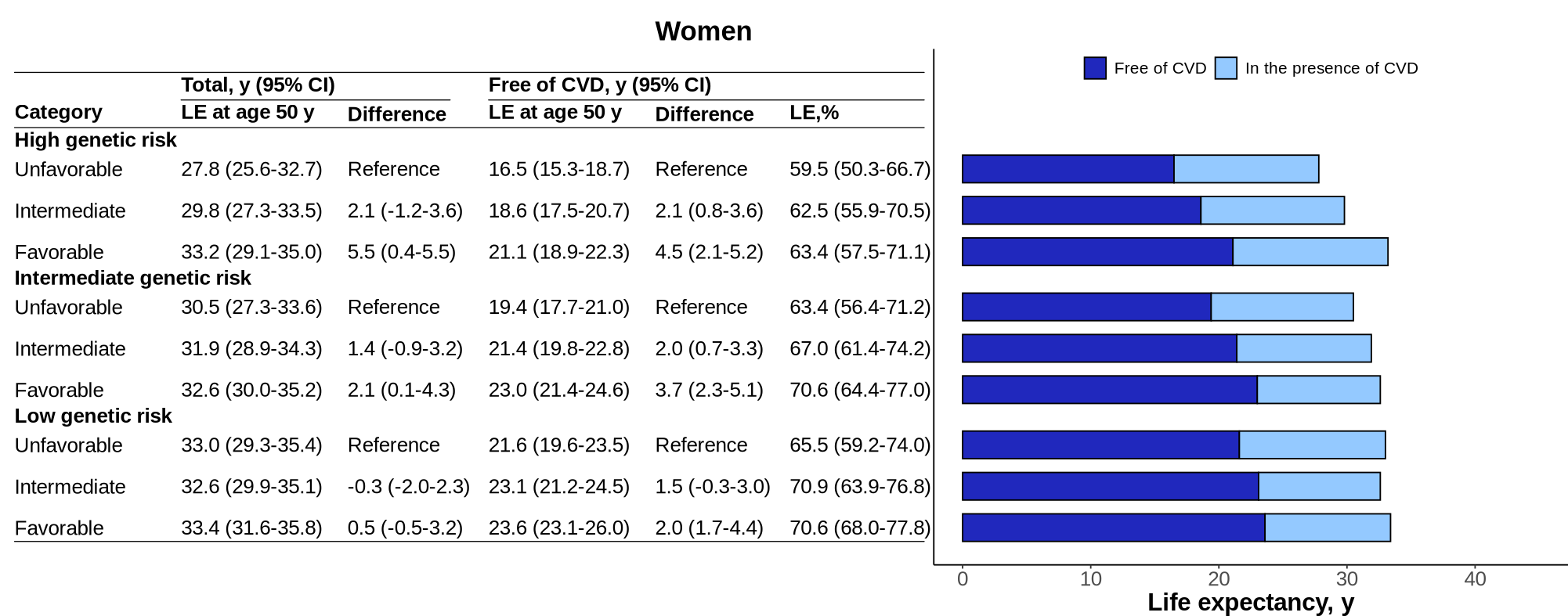

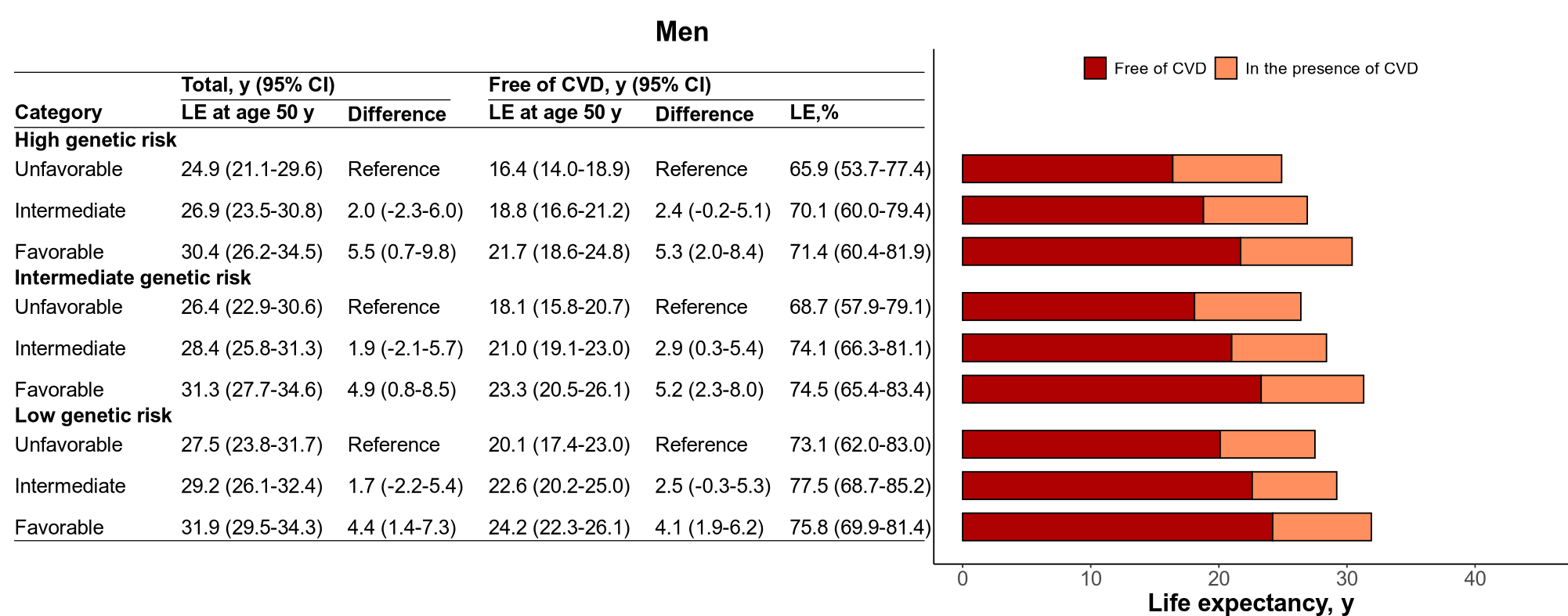
**

**Fig S14. Life expectancy at age 50 with and without cardiovascular disease according to joint categories of genetic risk and lifestyles in the testing set**

CI, confidence interval; CVD, cardiovascular disease; LE, life expectancy.

The genetic risk and lifestyles were categorized in the same way as in Table 2.

**
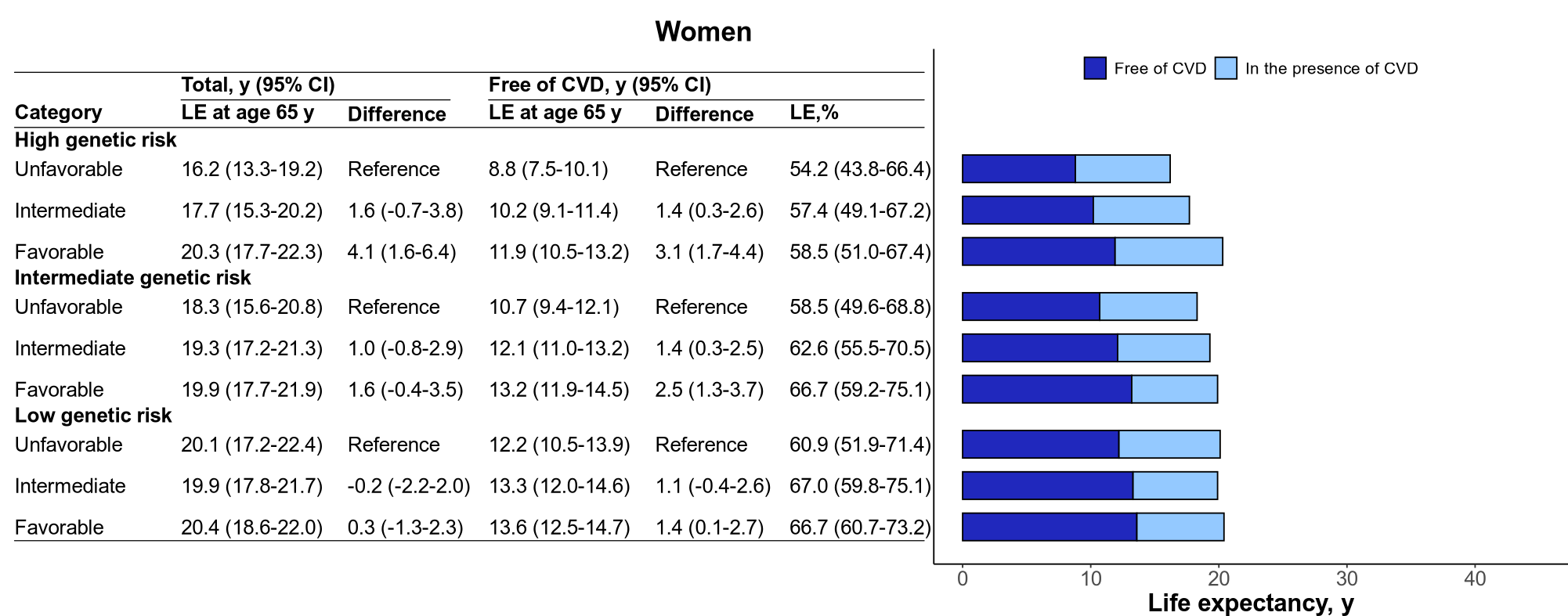

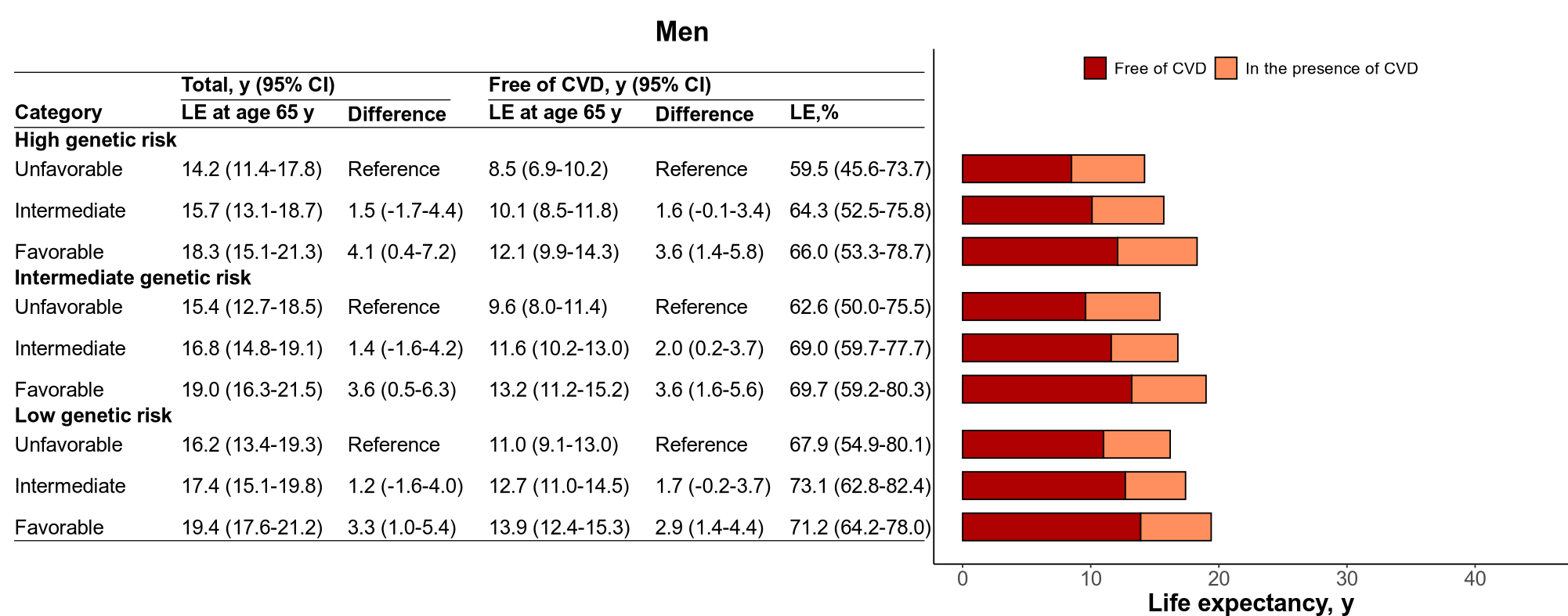
**

**Fig S15. Life expectancy at age 65 with and without cardiovascular disease according to joint categories of genetic risk and lifestyles in the testing set**

CI, confidence interval; CVD, cardiovascular disease; LE, life expectancy.

The genetic risk and lifestyles were categorized in the same way as in Table 2.

**Reference**

1. Zhu N, Yu C, Guo Y, Bian Z, Han Y, Yang L, et al. Adherence to a healthy lifestyle and all-cause and cause-specific mortality in Chinese adults: a 10-year prospective study of 0.5 million people. Int J Behav Nutr Phys Act. 2019;16(1):98.

2. Turnbull I, Clarke R, Wright N, Guo Y, Kartsonaki C, Pei P, et al. Diagnostic accuracy of major stroke types in Chinese adults: A clinical adjudication study involving 40,000 stroke cases. Lancet Reg Health West Pac. 2022;21:100415.

3. China TWCotRoCHaDi. Report on Cardiovascular Health and Diseases in China 2022: an Updated Summary. Chinese Circulation Journal. 2023;38(6).

4. Sollis E, Mosaku A, Abid A, Buniello A, Cerezo M, Gil L, et al. The NHGRI-EBI GWAS Catalog: knowledgebase and deposition resource. Nucleic Acids Res. 2023;51(D1):D977-d85.

5. Mak TSH, Porsch RM, Choi SW, Zhou X, Sham PC. Polygenic scores via penalized regression on summary statistics. Genet Epidemiol. 2017;41(6):469-80.

6. Ge T, Chen CY, Ni Y, Feng YA, Smoller JW. Polygenic prediction via Bayesian regression and continuous shrinkage priors. Nat Commun. 2019;10(1):1776.

7. Sun D, Ding Y, Yu C, Sun D, Pang Y, Pei P, et al. Joint impact of polygenic risk score and lifestyles on early- and late-onset cardiovascular diseases. Nature human behaviour. 2024;8(9):1810-8.

8. Patel AP, Wang M, Ruan Y, Koyama S, Clarke SL, Yang X, et al. A multi-ancestry polygenic risk score improves risk prediction for coronary artery disease. Nature medicine. 2023;29(7):1793-803.

9. Willer CJ, Li Y, Abecasis GR. METAL: fast and efficient meta-analysis of genomewide association scans. Bioinformatics (Oxford, England). 2010;26(17):2190-1.

10. Koyama S, Ito K, Terao C, Akiyama M, Horikoshi M, Momozawa Y, et al. Population-specific and trans-ancestry genome-wide analyses identify distinct and shared genetic risk loci for coronary artery disease. Nat Genet. 2020;52(11):1169-77.

11. Sakaue S, Kanai M, Tanigawa Y, Karjalainen J, Kurki M, Koshiba S, et al. A cross-population atlas of genetic associations for 220 human phenotypes. Nat Genet. 2021;53(10):1415-24.

12. Low SK, Takahashi A, Ebana Y, Ozaki K, Christophersen IE, Ellinor PT, et al. Identification of six new genetic loci associated with atrial fibrillation in the Japanese population. Nat Genet. 2017;49(6):953-8.

13. Ishigaki K, Akiyama M, Kanai M, Takahashi A, Kawakami E, Sugishita H, et al. Large-scale genome-wide association study in a Japanese population identifies novel susceptibility loci across different diseases. Nat Genet. 2020;52(7):669-79.

14. Kim YJ, Moon S, Hwang MY, Han S, Jang HM, Kong J, et al. The contribution of common and rare genetic variants to variation in metabolic traits in 288,137 East Asians. Nat Commun. 2022;13(1):6642.

15. Sun Q, Yu D, Fan J, Yu C, Guo Y, Pei P, et al. Healthy lifestyle and life expectancy at age 30 years in the Chinese population: an observational study. The Lancet Public health. 2022;7(12):e994-e1004.

16. Group CKBC. Healthy lifestyle and life expectancy free of major chronic diseases at age 40 in China. Nature human behaviour. 2023.

17. Barendregt JJ. The effect size in uncertainty analysis. Value Health. 2010;13(4):388-91.

18. Fan, Z. Poisson Regression. STATS 200: Introduction to Statistical Inference, Stanford University. <https://web.stanford.edu/class/archive/stats/stats200/stats200.1172/Lecture27.pdf>. Accessed 5 March 2025.

19. Aragam KG, Jiang T, Goel A, Kanoni S, Wolford BN, Atri DS, et al. Discovery and systematic characterization of risk variants and genes for coronary artery disease in over a million participants. Nat Genet. 2022;54(12):1803-15.

20. Mishra A, Malik R, Hachiya T, Jurgenson T, Namba S, Posner DC, et al. Stroke genetics informs drug discovery and risk prediction across ancestries. Nature. 2022;611(7934):115-23.

21. Roselli C, Chaffin MD, Weng LC, Aeschbacher S, Ahlberg G, Albert CM, et al. Multi-ethnic genome-wide association study for atrial fibrillation. Nat Genet. 2018;50(9):1225-33.

22. Levin MG, Tsao NL, Singhal P, Liu C, Vy HMT, Paranjpe I, et al. Genome-wide association and multi-trait analyses characterize the common genetic architecture of heart failure. Nat Commun. 2022;13(1):6914.

23. Evangelou E, Warren HR, Mosen-Ansorena D, Mifsud B, Pazoki R, Gao H, et al. Genetic analysis of over 1 million people identifies 535 new loci associated with blood pressure traits. Nat Genet. 2018;50(10):1412-25.

24. Graham SE, Clarke SL, Wu KH, Kanoni S, Zajac GJM, Ramdas S, et al. The power of genetic diversity in genome-wide association studies of lipids. Nature. 2021;600(7890):675-9.

25. Mahajan A, Taliun D, Thurner M, Robertson NR, Torres JM, Rayner NW, et al. Fine-mapping type 2 diabetes loci to single-variant resolution using high-density imputation and islet-specific epigenome maps. Nat Genet. 2018;50(11):1505-13.
